# Supplementary material for: CD8+ tissue-resident memory T cells induce oral lichen planus erosion via cytokine network
Source: eLife. 2023 Aug 9;12:e83981. doi: 10.7554/eLife.83981 (PMC10465124; doi:10.7554/eLife.83981)
Supplement: Figure 1—source code 1. [file elife-83981-fig1-code1.zip › 05-10-2022-RA-eLife-83981/Figure 1source code 1 related to Figure 1 A and B.docx]

library(Seurat)

library(SeuratData)

library(patchwork)

library(dplyr)

library(ggplot2)

setwd('F:/data/olp_stsc/analysis_data')

d10x.olp1 <- Read10X("./sc_file/EOLP-1/")

d10x.olp2 <- Read10X("./sc_file/EOLP-2/")

d10x.olp3 <- Read10X("./sc_file/NOLP-1/")

d10x.olp4 <- Read10X("./sc_file/NOLP-2/")

d10x.olp5 <- Read10X("./sc_file/NOLP-3/")

olp1 <- CreateSeuratObject(counts = d10x.olp1, project = "olp1", min.cells = 3, min.features = 200)

olp2 <- CreateSeuratObject(counts = d10x.olp2, project = "olp2", min.cells = 3, min.features = 200)

olp3 <- CreateSeuratObject(counts = d10x.olp3, project = "olp3", min.cells = 3, min.features = 200)

olp4 <- CreateSeuratObject(counts = d10x.olp4, project = "olp4", min.cells = 3, min.features = 200)

olp5 <- CreateSeuratObject(counts = d10x.olp5, project = "olp5", min.cells = 3, min.features = 200)

olp.merge <- merge(olp1, y = c(olp2, olp3,olp4,olp5), add.cell.ids = c("olp1", "olp2", "olp3",'olp4','olp5'), project = "olp")

tail(colnames(olp.merge))

table(olp.merge$orig.ident)

##根据样本分组colnames(olp.merge)

t<-colnames(olp.merge)

a <- substr(t,1,4)

b <- substr(t,23,23)

t<-paste(a,b, sep = "_")

t

table(t)

olp.merge <- AddMetaData(object=olp.merge, metadata=data.frame(gemgroup=t, row.names=rownames(olp.merge@meta.data)))

head(olp.merge@meta.data, 10)

table(olp.merge@meta.data[["gemgroup"]])

olp.merge[["percent.mt"]] <- PercentageFeatureSet(olp.merge, pattern = "^MT-")

head(olp.merge@meta.data)

col.num <- length(unique(olp.merge@meta.data$gemgroup))

Idents(olp.merge)<-'gemgroup'

violin <- VlnPlot(olp.merge,

features = c("nFeature_RNA", "nCount_RNA", "percent.mt"),

cols =rainbow(col.num),

pt.size = 0,

ncol = 3) +

theme(axis.title.x=element_blank(), axis.text.x=element_blank(), axis.ticks.x=element_blank())

violin

dim(olp.merge)#26164 47565

table(olp.merge$gemgroup)

#olp1_1 olp2_1 olp3_1 olp4_1 olp5_1

#8158 11631 11863 7590 8323

olp.merge <- subset(olp.merge, subset = nFeature_RNA<7500 & nCount_RNA > 500 & percent.mt < 25)

dim(olp.merge) #26164 46377

table(olp.merge@meta.data[["gemgroup"]])

#olp1_1 olp2_1 olp3_1 olp4_1 olp5_1

#7890 11545 11292 7435 8215

N1<-subset(olp.merge,gemgroup=='olp1_1')

N2<-subset(olp.merge,gemgroup=='olp2_1')

DK1<-subset(olp.merge,gemgroup=='olp3_1')

DK2<-subset(olp.merge,gemgroup=='olp4_1')

YK1<-subset(olp.merge,gemgroup=='olp5_1')

######To perform integration using the pearson residuals calculated above, we use the function after selecting a list of informative features using :PrepSCTIntegration()SelectIntegrationFeatures()

N1 <- SCTransform(N1, vst.flavor = "v2", verbose = FALSE) %>%

RunPCA(npcs = 30, verbose = FALSE)

N2 <- SCTransform(N2, vst.flavor = "v2", verbose = FALSE) %>%

RunPCA(npcs = 30, verbose = FALSE)

DK1 <- SCTransform(DK1, vst.flavor = "v2", verbose = FALSE) %>%

RunPCA(npcs = 30, verbose = FALSE)

DK2 <- SCTransform(DK2, vst.flavor = "v2", verbose = FALSE) %>%

RunPCA(npcs = 30, verbose = FALSE)

YK1 <- SCTransform(YK1, vst.flavor = "v2", verbose = FALSE) %>%

RunPCA(npcs = 30, verbose = FALSE)

olp.list <- list(N1=N1,N2=N2,DK1=DK1,DK2=DK2,YK1=YK1)

features <- SelectIntegrationFeatures(object.list = olp.list, nfeatures = 3000)

olp.list <- PrepSCTIntegration(object.list = olp.list, anchor.features = features)

#To integrate the two datasets, we use the function, which takes a list of Seurat objects as input, and use these anchors to integrate the two datasets together with .FindIntegrationAnchors()IntegrateData()

olp.anchors <- FindIntegrationAnchors(object.list = olp.list, normalization.method = "SCT",

anchor.features = features)#100% elapsed=01h 14m 40s

save(olp.anchors,file='./RData/20230411_anchors.RData')

load('./RData/20230411_anchors.RData')

olp.combined.sct <- IntegrateData(anchorset = olp.anchors, normalization.method = "SCT")

load("F:/data/olp_stsc/analysis_data/RData/20230411_SCT_olp.rdata")

#Perform an integrated analysis

olp.combined.sct@meta.data$lesion<-NA

olp.combined.sct@meta.data$lesion[which(olp.combined.sct@meta.data$gemgroup %in% c('olp1_1','olp2_1'))]<-"EOLP"

olp.combined.sct@meta.data$lesion[which(olp.combined.sct@meta.data$gemgroup %in% c('olp3_1','olp4_1','olp5_1'))]<-"NOLP"

table(olp.combined.sct@meta.data$lesion,olp.combined.sct@meta.data$gemgroup)

olp.combined.sct@meta.data$sample<-NA

olp.combined.sct@meta.data$sample[which(olp.combined.sct@meta.data$gemgroup %in% c('olp1_1'))]<-"EOLP_1"

olp.combined.sct@meta.data$sample[which(olp.combined.sct@meta.data$gemgroup %in% c('olp2_1'))]<-"EOLP_2"

olp.combined.sct@meta.data$sample[which(olp.combined.sct@meta.data$gemgroup %in% c('olp3_1'))]<-"NOLP_1"

olp.combined.sct@meta.data$sample[which(olp.combined.sct@meta.data$gemgroup %in% c('olp4_1'))]<-"NOLP_2"

olp.combined.sct@meta.data$sample[which(olp.combined.sct@meta.data$gemgroup %in% c('olp5_1'))]<-"NOLP_3"

olp.combined.sct@meta.data$sample<-factor(olp.combined.sct@meta.data$sample,levels=c("EOLP_1","EOLP_2","NOLP_1","NOLP_2","NOLP_3"))

table(olp.combined.sct@meta.data$sample,olp.combined.sct@meta.data$gemgroup)

olp<-olp.combined.sct

rm(olp.combined.sct)

DefaultAssay(olp) <- "integrated"

olp <- RunPCA(olp, verbose = FALSE)

olp <- RunUMAP(olp, reduction = "pca", dims = 1:15, verbose = FALSE)

olp <- RunTSNE(olp, reduction = "pca", dims = 1:15, verbose = FALSE)

olp <- FindNeighbors(olp, reduction = "pca", dims = 1:15)

olp <- FindClusters(olp, resolution = 1.8)

table(olp@meta.data[["gemgroup"]])

DimPlot(olp, group.by = c("sample"), reduction = "umap",combine = FALSE)

DimPlot(olp, group.by = c("lesion"), reduction = "umap",combine = FALSE)

DimPlot(olp, reduction = "umap", group.by = "integrated_snn_res.1.8", label = TRUE)#, repel = TRUE

##############cell types annotations###########

DefaultAssay(olp) <- "SCT"

#T cells 16

FeaturePlot(olp, features = c("CD2","CD3D","CD3E","CD3G"),label = T)

FeaturePlot(olp, reduction='tsne',features = c("CD2","CD3D","CD3E","CD3G"),label = T)

#NK cells

FeaturePlot(olp, features = c("GNLY","NKG7",'KLRF1','KLRC1'),label = T)

#B cells

FeaturePlot(olp, features = c("CD19","CD79A","BLNK","FCRL5",

'CD24','IGHG1' ),label = T,repel = F)

#b

FeaturePlot(olp, features = c('CD27','FCRL5','CD79A','CD19'),label = T,repel = F)

#Plasma

FeaturePlot(olp, features = c('CD19','CD24' ,'IGHG1', 'IGHA1', 'SDC1', 'CD79A'),label = T,repel = F)

#Macrophages

FeaturePlot(olp, features = c("CD14","CD163","CD68","FCGR2A","CSF1R"),label = T)

#Dentritic cells

FeaturePlot(olp, features = c("CD40","CD80","CD83","CCR7"),label = T)

#mast cells

FeaturePlot(olp, features = c("CMA1","MS4A2","TPSAB1","TPSB2"),label = T)

#neutrophil

FeaturePlot(olp, features = c("CXCL8","SOD2"),label = T)

#Epithelium

FeaturePlot(olp, features = c("KRT6B","KRT15","KRT6C","SFN","KRT14"),label = T)#"KRTCAP3"

FeaturePlot(olp, features = c("KRT5","S100A2","KRT15","SPRR1B","KRT14",'SFN'),label = T)

#Endothelial cells

FeaturePlot(olp, features = c("PECAM1","VWF","ENG",'SELE'),label = T)

#Fibroblast

FeaturePlot(olp, features = c("FAP","PDPN","COL1A2","DCN","COL3A1","COL6A1"),label = T)

#myocytes

FeaturePlot(olp, features = c("ACTA1",'ACTA2',"ACTN2","MYL2","MYH2"),label = T)

FeaturePlot(olp, features = c('MPZ','PMP2','PLP1'),label = T)

markers<-c('CD2','CD3D','CD3E','CD3G',#T

'GNLY','NKG7',#NK

'CD79A','IGHG1',#B/Plasma

"CMA1","MS4A2","TPSAB1",

"LYZ",'HLA-DRB1', 'HLA-DRA',#Myeloid

'PECAM1','VWF','ENG','SELE',#Endo

'KRT5','S100A2','KRT15','SPRR18',#Epi

'COL1A2','DCN','COL3A1','COL6A1',#Fibrorlast

'PMP2','PLP1'#Myocyte

)

DotPlot(olp, features=markers)+ coord_flip()

DefaultAssay(olp) <- "SCT"

Idents(olp)<-"integrated_snn_res.1.8"

marker41 <- FindMarkers(olp, assay = "SCT",ident.1 = "41",only.pos = TRUE, min.pct = 0.25, logfc.threshold = 0.25,recorrect_umi = FALSE)

marker38 <- FindMarkers(olp, assay = "SCT",ident.1 = "38",only.pos = TRUE, min.pct = 0.25, logfc.threshold = 0.25,recorrect_umi = FALSE)

marker39 <- FindMarkers(olp, assay = "SCT",ident.1 = "39",only.pos = TRUE, min.pct = 0.25, logfc.threshold = 0.25,recorrect_umi = FALSE)

marker32 <- FindMarkers(olp, assay = "SCT",ident.1 = "32",only.pos = TRUE, min.pct = 0.25, logfc.threshold = 0.25,recorrect_umi = FALSE)

marker44 <- FindMarkers(olp, assay = "SCT",ident.1 = "44",only.pos = TRUE, min.pct = 0.25, logfc.threshold = 0.25,recorrect_umi = FALSE)

olp@meta.data$celltype<-NA

olp@meta.data$celltype[which(olp@meta.data$integrated_snn_res.1.8 %in% c(15,16,19,3,6,11,4,2,24,9,7,8,34,23,1,17,10,0,18,42,40))]<-"T/NK cells"

olp@meta.data$celltype[which(olp@meta.data$integrated_snn_res.1.8 %in% c(5,41,39))]<-"B/Plasma cells"

olp@meta.data$celltype[which(olp@meta.data$integrated_snn_res.1.8 %in% c(21,33))]<-"Mast cells"

olp@meta.data$celltype[which(olp@meta.data$integrated_snn_res.1.8 %in% c(43,28,26,13,31))]<-"Myeloid cells"#

olp@meta.data$celltype[which(olp@meta.data$integrated_snn_res.1.8 %in% c(45,44,29))]<-"Epithelium cells"#

olp@meta.data$celltype[which(olp@meta.data$integrated_snn_res.1.8 %in% c( 35,36,14,22))]<-"Endothelial cells"#

olp@meta.data$celltype[which(olp@meta.data$integrated_snn_res.1.8 %in% c(20,25,32,38,12,30,37,27))]<-"Fibroblast cells"#

olp@meta.data$celltype[which(olp@meta.data$integrated_snn_res.1.8 %in% c(46))]<-"Neural cells"

table(olp@meta.data$celltype,olp@meta.data$integrated_snn_res.1.8)

DimPlot(olp, reduction = "umap", group.by = "celltype", label = F)#, repel = TRUE

DimPlot(olp, reduction = "tsne", group.by = "celltype", label = TRUE)#, repel = TRUE

DimPlot(olp, reduction = "umap", group.by = "integrated_snn_res.1.8", label = TRUE)#, repel = TRUE

DimPlot(olp, reduction = "tsne", group.by = "integrated_snn_res.1.8", label = TRUE)#, repel = TRUE

DimPlot(olp, reduction = "umap",group.by = "celltype", split.by = "lesion")

DimPlot(olp, reduction = "tsne",group.by = "celltype", split.by = "lesion")

DimPlot(olp, reduction = "umap",group.by = "tcell_type")

Idents(olp)<-"celltype"

DotPlot(olp, features=markers)+ coord_flip()

####Fig

library(Seurat)

library(SeuratData)

library(patchwork)

library(dplyr)

library(ggplot2)

####ALL CELLS####################

load("F:/data/qmf/olp/RData/20230411_resct_olp.RData")

##cellratio

library(ggsci)

library(ggplot2)

library(RColorBrewer)

mycolor<-colorRampPalette((pal_npg("nrc")(9)))(47)

##Fig 1a

DimPlot(olp, reduction = "umap", group.by = "integrated_snn_res.1.8", label = TRUE,cols = mycolor)#, repel = TRUE

mycolor<-pal_npg("nrc", alpha = 0.7)(8)

##Fig 1b

DimPlot(olp, reduction = "umap", group.by = "celltype", label = F,cols = mycolor)#, repel = TRUE

table(olp$celltype,olp$sample)

Cellratio <- prop.table(table(olp$celltype,olp$sample), margin = 2)

Cellratio <- as.data.frame(Cellratio)

colourCount = length(unique(Cellratio$Var1))

Cellratio$Var1<- factor(Cellratio$Var1, level=c('T/NK cells','B/Plasma cells',"Mast cells", 'Myeloid cells',

'Epithelium cells','Endothelial cells','Fibroblast cells',

'Neural cells'))

colnames(Cellratio)[1] <- 'Celltype'

write.csv(Cellratio,file = 'F:/data/olp_stsc/analysis_data/table/fig1c.csv')

#Fig 1c

ggplot(Cellratio) +

geom_bar(aes(x =Var2, y= round(Freq,4), fill = Celltype ),stat = "identity",width = 0.7,size = 0.5,colour = '#222222')+

theme_classic() +

labs(x='Sample',y = 'Ratio')+scale_fill_npg(alpha=0.7)+#+scale_fill_brewer(palette = "Set2")+

theme(axis.text = element_text(size = 15, face = 'bold'))

Cellratio <- prop.table(table(olp$sample,olp$celltype), margin = 2)

Cellratio <- as.data.frame(Cellratio)

colourCount = length(unique(Cellratio$Var1))

colnames(Cellratio)[1] <- 'Sample'

write.csv(Cellratio,file = 'F:/data/olp_stsc/analysis_data/table/fig1d.csv')

#Fig 1d

ggplot(Cellratio) +

geom_bar(aes(x =Var2, y= round(Freq,4), fill = Sample ),stat = "identity",width = 0.7,size = 0.5,colour = '#222222')+

theme_classic() +

labs(x='Celltype',y = 'Ratio')+scale_fill_npg(alpha=0.7)+#+scale_fill_brewer(palette = "Set2")+

theme(axis.text = element_text(size = 15, face = 'bold'))+coord_flip()
