# Supplementary material for: CD8+ tissue-resident memory T cells induce oral lichen planus erosion via cytokine network
Source: eLife. 2023 Aug 9;12:e83981. doi: 10.7554/eLife.83981 (PMC10465124; doi:10.7554/eLife.83981)
Supplement: Figure 1—figure supplement 1—source code 1. [file elife-83981-fig1-figsupp1-code1.zip › 05-10-2022-RA-eLife-83981/Figure 1supplement 1 source code 1 related to Figure 1supplement 1C D and E.docx]

library(Seurat)

library(xtable)

library(reshape2)

Idents(olp)<-olp@meta.data$celltype

table(olp$celltype)

####Epi####################

olp.MYE<-subset(olp,idents = "Myeloid cells")

Idents(olp.MYE)<-olp.MYE@meta.data$sample

DimPlot(olp.MYE, reduction = "umap", label = F,pt.size = 0.5)

DefaultAssay(olp.MYE) <- "integrated"

olp.MYE <- RunPCA(olp.MYE, verbose = FALSE)

olp.MYE <- RunUMAP(olp.MYE, reduction = "pca", dims = 1:10, verbose = FALSE)

olp.MYE <- RunTSNE(olp.MYE, reduction = "pca", dims = 1:10, verbose = FALSE)

olp.MYE <- FindNeighbors(olp.MYE, reduction = "pca", dims = 1:10)

olp.MYE <- FindClusters(olp.MYE, resolution = 0.1)

DimPlot(olp.MYE, reduction = "umap", label = TRUE)

DimPlot(olp.MYE, reduction = "umap", label = TRUE,group.by = 'lesion')

DefaultAssay(olp.MYE) <- "SCT"

#Macrophages

FeaturePlot(olp, features = c("CD14","CD163","CD68","FCGR2A","CSF1R"),label = T)

#Dentritic cells

FeaturePlot(olp, features = c("CD40","CD80","CD83","CCR7"),label = T)

#mast cells

FeaturePlot(olp, features = c("CMA1","MS4A2","TPSAB1","TPSB2"),label = T)

#neutrophil

FeaturePlot(olp, features = c("CXCL8","SOD2"),label = T)

features<-c("CD14","CD163","CD68","FCGR2A","CSF1R",

"CD40","CD80","CD83","CCR7",

"CXCL8","SOD2")

immune_cellmarker <- c('S100A8','CXCL8','SOD2','NAMPT',#Neutrophil

"CD14","CD163","CD68","FCGR2A","CSF1R",#Macrophage

#'TPSAB1','TPSB2','CPA3','HPGDS',#Mast

'HLA-DRA','HLA-DPB1','CST3','HLA-DPA1',#mDC

'PTGDS','SOX4','GZMB','IRF7'#pDC

)

DefaultAssay(olp.MYE) <- "SCT"

DotPlot(olp.MYE,features = features,scale =F)+ coord_flip()

DotPlot(olp.MYE,features = features,scale =T)+ coord_flip()

DefaultAssay(olp.MYE) <- "RNA"

DotPlot(olp.MYE,features = immune_cellmarker,scale = T)+coord_flip()

DefaultAssay(olp.MYE) <- "SCT"

DotPlot(olp.MYE,features = immune_cellmarker,scale = T)+coord_flip()

DefaultAssay(olp.MYE) <- "RNA"

Idents(olp.MYE)<-'integrated_snn_res.0.1'

DimPlot(olp.MYE, reduction = "umap",label = T)+NoLegend()

all.markers <- FindAllMarkers(olp.MYE, only.pos = TRUE, min.pct = 0.25, logfc.threshold = 0.25)

top50 <- all.markers %>% group_by(cluster) %>% top_n(50,avg_log2FC)

top3 <- all.markers %>% group_by(cluster) %>% top_n(3,avg_log2FC)

setwd('F:/data/olp_stsc/analysis_data')

write.table(top50,"./table/20230424_olp.mye_top50_0.1.csv",col.names=T,row.names=F,sep=",")

olp.MYE@meta.data$celltype<-NA

olp.MYE@meta.data$celltype[which(olp.MYE@meta.data$integrated_snn_res.0.1 %in% c(0,2))]<-"Neutrophil"

olp.MYE@meta.data$celltype[which(olp.MYE@meta.data$integrated_snn_res.0.1 %in% c(1))]<-"Macrophage"

olp.MYE@meta.data$celltype[which(olp.MYE@meta.data$integrated_snn_res.0.1 %in% c(3))]<-"mDC"

olp.MYE@meta.data$celltype[which(olp.MYE@meta.data$integrated_snn_res.0.1 %in% c(4))]<-"pDC"

DotPlot(olp.MYE, features=immune_cellmarker,group.by = "celltype",scale = T)+ coord_flip()

DimPlot(olp.MYE, reduction = "umap", group.by = "celltype", label = TRUE)#, repel = TRUE

Idents(olp.MYE)<-'celltype'

#setwd('F:/data/olp_stsc/analysis_data')

#load( './Rdata/20230424_olp.MYE_label.RData')

mycolor<-colorRampPalette((pal_npg("nrc")(5)))(5)#

#figs1c

DimPlot(olp.MYE, reduction = "umap", group.by = 'integrated_snn_res.0.1',split.by = 'sample',cols = mycolor,label = T)

Idents(olp.MYE)<-'celltype'

table(olp.MYE$celltype)

DefaultAssay(olp.MYE) <- "RNA"

olp.MYE.markers <- FindAllMarkers(olp.MYE,only.pos = T,min.pct = 0.1,logfc.threshold = 0.25)

top10 <- olp.MYE.markers%>%group_by(cluster)%>%top_n(n=10,wt=avg_log2FC)

#figs1d

DoHeatmap(olp.MYE,features = top10$gene,slot = 'data')+scale_fill_gradientn(colors = c("white","grey","firebrick3"))+NoLegend()

Idents(olp)<-olp@meta.data$celltype

table(Idents(olp))

olp.MYE_all<-subset(olp,idents = c("Myeloid cells",'Mast cells') )

b<-olp.MYE@meta.data

table(b$celltype)

olp.MYE_all@meta.data$celltype2<-olp.MYE_all@meta.data$celltype

olp.MYE_all@meta.data$celltype2[which(rownames(olp.MYE_all@meta.data) %in% rownames(b))]<-b$celltype

table(olp.MYE_all$celltype2)

mycolor<-colorRampPalette((pal_npg("nrc")(8)))(8)

table(olp.MYE_all$celltype2)

table(olp.MYE_all$celltype2,olp.MYE_all$celltype)

DimPlot(olp.MYE_all, reduction = "umap",group.by = "celltype2",cols = mycolor)

Cellratio <- prop.table(table(olp.MYE_all$celltype2,olp.MYE_all$sample), margin = 2)

Cellratio <- as.data.frame(Cellratio)

colourCount = length(unique(Cellratio$Var1))

Cellratio$Var1<- factor(Cellratio$Var1, level=c('Neutrophil','Macrophage','pDC','mDC','Mast cells'))

colnames(Cellratio)[1] <- 'Celltype'

#figs1a

write.csv(Cellratio,file = 'F:/data/olp_stsc/analysis_data/table/figs1a.csv')

ggplot(Cellratio) +

geom_bar(aes(x =Var2, y= round(Freq,4), fill = Celltype ),stat = "identity",width = 0.7,size = 0.5,colour = '#222222')+

theme_classic() +

labs(x='lesion',y = 'Ratio')+scale_fill_npg(alpha=0.7)+

gene <- bitr(gene,fromType = 'SYMBOL',toType = 'ENTREZID',OrgDb = GO_database)

GO<-enrichGO(gene$ENTREZID,

OrgDb = GO_database,

keyType = "ENTREZID",

ont = "ALL",

pvalueCutoff = 0.05,

qvalueCutoff = 0.05,

readable = T)

#Figs1e

dotplot(GO,showCategory = 15
