# Supplementary material for: CD8+ tissue-resident memory T cells induce oral lichen planus erosion via cytokine network
Source: eLife. 2023 Aug 9;12:e83981. doi: 10.7554/eLife.83981 (PMC10465124; doi:10.7554/eLife.83981)
Supplement: Figure 2—source code 1. [file elife-83981-fig2-code1.zip › 05-10-2022-RA-eLife-83981/Figure 2source code 1 related to Figure 2 A B D E F and G.docx]

library(Seurat)

library(xtable)

library(reshape2)

Idents(olp)<-olp@meta.data$celltype

table(olp$celltype)

####Epi####################

olp.TNK<-subset(olp,idents = "T/NK cells")

Idents(olp.TNK)<-olp.TNK@meta.data$sample

DimPlot(olp.TNK, reduction = "umap", label = F,pt.size = 0.5)

DefaultAssay(olp.TNK) <- "integrated"

olp.TNK <- RunPCA(olp.TNK, verbose = FALSE)

olp.TNK <- RunUMAP(olp.TNK, reduction = "pca", dims = 1:10, verbose = FALSE)

olp.TNK <- RunTSNE(olp.TNK, reduction = "pca", dims = 1:10, verbose = FALSE)

olp.TNK <- FindNeighbors(olp.TNK, reduction = "pca", dims = 1:10)

olp.TNK <- FindClusters(olp.TNK, resolution = 0.5)

DimPlot(olp.TNK, reduction = "umap", label = TRUE)

tfeatures<-c('CD2','CD3E','CD3D' , 'CD3G', 'CD4', 'CD5', 'CD7',

'CD8A', 'CD8B','CD27', 'CD28',

'CD69', 'CD44', 'CD103','ITGAE', 'CD49a','ITGA1', 'LFA-1','ITGB2', 'CD45RO','UCHL1', 'CCR7', 'S1PR1',

'KLRB1','KLRC1','CD16','CD56','NCAM1','CD94','KLRD1' )

DefaultAssay(olp.TNK) <- "RNA"

DotPlot(olp.TNK,features = tfeatures,scale = F)+ coord_flip()

DotPlot(olp.TNK,features = tfeatures)

olp.TNK@meta.data$celltype<-NA

olp.TNK@meta.data$celltype[which(olp.TNK@meta.data$integrated_snn_res.1 %in% c(2,3,4,5,8,9,18,17,20))]<-"CD4+ T"

olp.TNK@meta.data$celltype[which(olp.TNK@meta.data$integrated_snn_res.1 %in% c(1,0,11,14,15,19))]<-"CD8+ T"

olp.TNK@meta.data$celltype[which(olp.TNK@meta.data$integrated_snn_res.1 %in% c(12,16))]<-"CD4+ CD8+ T"

olp.TNK@meta.data$celltype[which(olp.TNK@meta.data$integrated_snn_res.1 %in% c(13,21,22))]<-"NK"#

olp.TNK@meta.data$celltype[which(olp.TNK@meta.data$integrated_snn_res.1 %in% c(6,10))]<-"CD8+ TRM"#

olp.TNK@meta.data$celltype[which(olp.TNK@meta.data$integrated_snn_res.1 %in% c(7))]<-"CD4+ CD8+ TRM"#

Idents(olp.TNK)<-'celltype'

mycolor<-colorRampPalette((pal_npg("nrc")(6)))(6)

#fig 2a

DimPlot(olp.TNK, reduction = "umap", group.by = 'celltype',cols = mycolor,label = T)

Idents(olp.TNK)<-'celltype'

olp.TNK.markers <- FindAllMarkers(olp.TNK,only.pos = T,min.pct = 0.1,logfc.threshold = 0.25)

top3 <- olp.TNK.markers%>%group_by(cluster)%>%top_n(n=3,wt=avg_log2FC)

tfeatures<-c('CD2','CD3E','CD3D' , 'CD3G', 'CD4', 'CD5', 'CD7',

'CD8A', 'CD8B','CD27', 'CD28',

'CD69', 'CD44', 'CD103','ITGAE', 'CD49a','ITGA1', 'LFA-1','ITGB2', 'CD45RO','UCHL1', 'CCR7', 'S1PR1',

'KLRB1','KLRC1','CD16','CD56','NCAM1','CD94','KLRD1' )

DefaultAssay(olp.TNK) <- "RNA"

#fig2b

DotPlot(olp.TNK,features = tfeatures,scale = F)+ coord_flip()

DefaultAssay(olp.TNK) <- "integrated"

#fig2c

table(olp.TNK$celltype,olp.TNK$lesion)

Cellratio <- prop.table(table(olp.TNK$celltype,olp.TNK$lesion), margin = 2)

Cellratio <- as.data.frame(Cellratio)

colourCount = length(unique(Cellratio$Var1))

Cellratio$Var1<- factor(Cellratio$Var1, level=c("CD4+ T",'CD8+ T',"CD4+ CD8+ T","CD8+ TRM","CD4+ CD8+ TRM","NK"))

colnames(Cellratio)[1] <- 'Celltype'

write.csv(Cellratio,file = 'F:/data/olp_stsc/analysis_data/table/fig2c.csv')

ggplot(Cellratio) +

geom_bar(aes(x =Var2, y= round(Freq,4), fill = Celltype ),stat = "identity",width = 0.7,size = 0.5,colour = '#222222')+

theme_classic() +

labs(x='lesion',y = 'Ratio')+scale_fill_npg(alpha=0.7)+

theme(axis.text = element_text(size = 15, face = 'bold'))

#fig 2d

DotPlot(olp.TNK,features = top3$gene,scale = F)+ coord_flip()

write.table(olp.TNK.markers,"./table/20230423_olp.TNK.markers0.csv",col.names=T,row.names=F,sep=",")

inf_features<-c('IL17','TNFSF14','TNFAIP3','TNF','TNFRSF4','TNFSF9','GZMA'

,'GZMB','GZMK', 'IFNG','PRF1')

DefaultAssay(olp.TNK) <- "RNA"

DotPlot(olp.TNK,features = inf_features,scale = F)+ coord_flip()

DefaultAssay(olp.TNK) <- "integrated"

#fig2e

DotPlot(olp.TNK,features = inf_features,scale = F)+ coord_flip()

table(Idents(olp.TNK))

CD8TRM<-subset(olp.TNK,idents = 'CD8+ TRM')

Idents(CD8TRM)<-'lesion'

table(Idents(CD8TRM))

CD8TRM.markers <- FindMarkers(CD8TRM,ident.1 = 'EOLP',ident.2 = 'NOLP',

only.pos = T,min.pct = 0.1,logfc.threshold = 0)

library(AnnotationDbi)

library(org.Hs.eg.db

library(clusterProfiler)

library(dplyr)

library(ggplot2)

gene<-CD8TRM.markers

colnames(gene)

gene <- subset(gene, p_val < 0.05 & avg_log2FC > 0)

gene<-rownames(gene)

gene

GO_database <- 'org.Hs.eg.db'

KEGG_database <- 'hsa'

gene <- bitr(gene,fromType = 'SYMBOL',toType = 'ENTREZID',OrgDb = GO_database)

GO<-enrichGO(gene$ENTREZID,

OrgDb = GO_database,

keyType = "ENTREZID",

ont = "ALL",

pvalueCutoff = 0.05,

qvalueCutoff = 0.05,

readable = T)

KEGG<-enrichKEGG(gene$ENTREZID,

organism = KEGG_database,

pvalueCutoff = 0.05,

qvalueCutoff = 0.05)

#fig2f

barplot(GO,showCategory = 40)

dotplot(GO,showCategory = 20)

###fig2g

library(Seurat)

library(monocle)

library(fda.usc)

library(dplyr)

library(patchwork)

library(DESeq2)

table(olp.TNK$gemgroup)

expr_matrix <- as(as.matrix(olp.TNK@assays$RNA@counts), 'sparseMatrix')

p_data <- olp.TNK@meta.data

f_data <- data.frame(gene_short_name = row.names(olp.TNK@assays$RNA@counts),row.names = row.names(olp.TNK@assays$RNA@counts))

pd <- new('AnnotatedDataFrame', data = p_data)

fd <- new('AnnotatedDataFrame', data = f_data)

cds <- newCellDataSet(expr_matrix,

phenoData = pd,

featureData = fd,

lowerDetectionLimit = 0.5,

expressionFamily = negbinomial.size())

cds <- estimateSizeFactors(cds)

cds <- estimateDispersions(cds)

cds <- detectGenes(cds, min_expr = 0.1)

Dim(cds)

print(head(fData(cds)))

expressed_genes <- row.names(subset(fData(cds),

num_cells_expressed >= 10))

length(expressed_genes)

disp_table <- dispersionTable(cds)

disp.genes <- subset(disp_table, mean_expression >= 0.01 & dispersion_empirical >= 1 * dispersion_fit)$gene_id

cds <- setOrderingFilter(cds, disp.genes)

plot_ordering_genes(cds)

cds <- reduceDimension(cds, max_components = 2,

method = 'DDRTree')

cds <- orderCells(cds)

table(cds$celltype)

cds$celltype<-factor(cds$celltype,level=c('CD8+ T','CD8+ TRM','CD4+ CD8+ T','CD4+ CD8+ TRM','CD4+ T', 'NK'))

plot_cell_trajectory(cds,color_by="Pseudotime", size=1,show_backbone=TRUE)

library(ggsci)

library(scales)

mycolor<-c("#B34EE6FF","#FF0000FF",'#C9F1FCFF','#13FB00FF','#FFD79CFF','#FFC900FF')

plot_cell_trajectory(cds,color_by="celltype", size=1,show_backbone=TRUE)+scale_color_manual(values = mycolor)
