# Supplementary material for: CD8+ tissue-resident memory T cells induce oral lichen planus erosion via cytokine network
Source: eLife. 2023 Aug 9;12:e83981. doi: 10.7554/eLife.83981 (PMC10465124; doi:10.7554/eLife.83981)
Supplement: Figure 2—figure supplement 1—source code 1. [file elife-83981-fig2-figsupp1-code1.zip › 05-10-2022-RA-eLife-83981/Figure 2supplement 1 source code 1 related to Figure 2supplement 1A B and C.docx]

library(Seurat)

library(xtable)

library(reshape2)

Idents(olp)<-olp@meta.data$celltype

table(olp$celltype)

####Epi####################

olp.TNK<-subset(olp,idents = "T/NK cells")

Idents(olp.TNK)<-olp.TNK@meta.data$sample

DimPlot(olp.TNK, reduction = "umap", label = F,pt.size = 0.5)

DefaultAssay(olp.TNK) <- "integrated"

olp.TNK <- RunPCA(olp.TNK, verbose = FALSE)

olp.TNK <- RunUMAP(olp.TNK, reduction = "pca", dims = 1:10, verbose = FALSE)

olp.TNK <- RunTSNE(olp.TNK, reduction = "pca", dims = 1:10, verbose = FALSE)

olp.TNK <- FindNeighbors(olp.TNK, reduction = "pca", dims = 1:10)

olp.TNK <- FindClusters(olp.TNK, resolution = 0.5)

DimPlot(olp.TNK, reduction = "umap", label = TRUE)

tfeatures<-c('CD2','CD3E','CD3D' , 'CD3G', 'CD4', 'CD5', 'CD7',

'CD8A', 'CD8B','CD27', 'CD28',

'CD69', 'CD44', 'CD103','ITGAE', 'CD49a','ITGA1', 'LFA-1','ITGB2', 'CD45RO','UCHL1', 'CCR7', 'S1PR1','KLRB1','KLRC1','CD16','CD56','NCAM1','CD94','KLRD1' )

DefaultAssay(olp.TNK) <- "RNA"

DotPlot(olp.TNK,features = tfeatures,scale = F)+ coord_flip()

DotPlot(olp.TNK,features = tfeatures)

olp.TNK@meta.data$celltype<-NA

olp.TNK@meta.data$celltype[which(olp.TNK@meta.data$integrated_snn_res.1 %in% c(2,3,4,5,8,9,18,17,20))]<-"CD4+ T"

olp.TNK@meta.data$celltype[which(olp.TNK@meta.data$integrated_snn_res.1 %in% c(1,0,11,14,15,19))]<-"CD8+ T"

olp.TNK@meta.data$celltype[which(olp.TNK@meta.data$integrated_snn_res.1 %in% c(12,16))]<-"CD4+ CD8+ T"

olp.TNK@meta.data$celltype[which(olp.TNK@meta.data$integrated_snn_res.1 %in% c(13,21,22))]<-"NK"#

olp.TNK@meta.data$celltype[which(olp.TNK@meta.data$integrated_snn_res.1 %in% c(6,10))]<-"CD8+ TRM"#

olp.TNK@meta.data$celltype[which(olp.TNK@meta.data$integrated_snn_res.1 %in% c(7))]<-"CD4+ CD8+ TRM"#

DotPlot(olp.TNK, features=tfeatures,group.by = "celltype")+ coord_flip()

DimPlot(olp.TNK, reduction = "umap", group.by = "celltype", label = TRUE)#, repel = TRUE

DefaultAssay(olp.TNK) <- "RNA"

DotPlot(olp.TNK,features = tfeatures,scale = F,group.by = "celltype")+ coord_flip()

Idents(olp.TNK)<-'celltype'

mycolor<-colorRampPalette((pal_npg("nrc")(9)))(23)

##fig s2a

DimPlot(olp.TNK, reduction = "umap", group.by = 'integrated_snn_res.1',split.by = 'sample',cols = mycolor,label = T)

Idents(olp.TNK)<-'integrated_snn_res.1'

table(olp.TNK$integrated_snn_res.1)

DefaultAssay(olp.TNK) <- "RNA"

olp.TNK.markers <- FindAllMarkers(olp.TNK,only.pos = T,min.pct = 0.1,logfc.threshold = 0.25)

top3 <- olp.TNK.markers%>%group_by(cluster)%>%top_n(n=3,wt=avg_log2FC)

#DefaultAssay(olp.TNK) <- "SCT"

#fig s2b

DoHeatmap(olp.TNK,features = top3$gene,slot = 'data')+scale_fill_gradientn(colors = c("white","grey","firebrick3"))+NoLegend()

Idents(olp.TNK)<-'celltype'

table(olp.TNK$celltype)

DefaultAssay(olp.TNK) <- "RNA"

olp.TNK.markers <- FindAllMarkers(olp.TNK,only.pos = T,min.pct = 0.1,logfc.threshold = 0.25)

top10 <- olp.TNK.markers%>%group_by(cluster)%>%top_n(n=10,wt=avg_log2FC)

##fig s2c

DoHeatmap(olp.TNK,features = top10$gene,slot = 'data')+scale_fill_gradientn(colors = c("white","grey","firebrick3"))+NoLegend()
