# Supplementary material for: CD8+ tissue-resident memory T cells induce oral lichen planus erosion via cytokine network
Source: eLife. 2023 Aug 9;12:e83981. doi: 10.7554/eLife.83981 (PMC10465124; doi:10.7554/eLife.83981)
Supplement: Supplementary file 1. [file elife-83981-supp1.docx]

**Supplementary Files**

**This file includes:**

**Supplementary File 1a, 1b and 1c**

**Supplementary File 1a. List of genes with statistically significant differences between EOLP and NEOLP.**

|  | p_val | avg_log2FC | pct.1 | pct.2 | p_val_adj |
| --- | --- | --- | --- | --- | --- |
| HSP90AA1 | 0 | 1.57662634 | 0.977 | 0.902 | 0 |
| HSPA6 | 0 | 1.54515426 | 0.5 | 0.257 | 0 |
| HSPA1A | 0 | 1.51369086 | 0.846 | 0.63 | 0 |
| HSP90AB1 | 0 | 1.43600437 | 0.895 | 0.775 | 0 |
| NFKBIA | 8.83E-295 | 1.31498831 | 0.613 | 0.428 | 2.31E-290 |
| HSPH1 | 0 | 1.29250753 | 0.682 | 0.411 | 0 |
| HSPD1 | 0 | 1.26424469 | 0.744 | 0.521 | 0 |
| DNAJB1 | 0 | 1.22205393 | 0.887 | 0.686 | 0 |
| PPP1R15A | 0 | 1.18206113 | 0.808 | 0.547 | 0 |
| SERPINH1 | 0 | 1.14486888 | 0.313 | 0.107 | 0 |
| ZFAND2A | 0 | 1.12839239 | 0.365 | 0.123 | 0 |
| DNAJA1 | 0 | 1.12286682 | 0.715 | 0.483 | 0 |
| HSPA1B | 0 | 1.10489988 | 0.813 | 0.63 | 0 |
| HSPA8 | 0 | 1.06492707 | 0.878 | 0.731 | 0 |
| DDIT4 | 0 | 1.01609399 | 0.653 | 0.417 | 0 |
| FTL | 0 | 1.00638217 | 0.909 | 0.771 | 0 |
| HSPB1 | 0 | 1.00177719 | 0.723 | 0.555 | 0 |
| FTH1 | 0 | 0.99122848 | 0.954 | 0.872 | 0 |
| CACYBP | 0 | 0.97404279 | 0.668 | 0.419 | 0 |
| BAG3 | 0 | 0.8557926 | 0.324 | 0.079 | 0 |
| RPS2 | 0 | 0.85511227 | 0.954 | 0.918 | 0 |
| RPS16 | 0 | 0.85216994 | 0.944 | 0.895 | 0 |
| RPL18A | 0 | 0.84814426 | 0.943 | 0.895 | 0 |
| TPT1 | 0 | 0.84785194 | 0.979 | 0.945 | 0 |
| JUN | 6.05E-279 | 0.82717871 | 0.809 | 0.64 | 1.58E-274 |
| UBC | 0 | 0.82543476 | 0.956 | 0.867 | 0 |
| SAT1 | 0 | 0.7994587 | 0.78 | 0.591 | 0 |
| HSPE1 | 1.68E-258 | 0.78685672 | 0.771 | 0.628 | 4.40E-254 |
| DUSP1 | 1.09E-234 | 0.77464085 | 0.738 | 0.587 | 2.85E-230 |
| RPL15 | 0 | 0.77252389 | 0.936 | 0.872 | 0 |
| RPS6 | 0 | 0.77118899 | 0.949 | 0.906 | 0 |
| RPS18 | 0 | 0.76795131 | 0.961 | 0.927 | 0 |
| RPL13 | 0 | 0.76211275 | 0.971 | 0.951 | 0 |
| RPL13A | 0 | 0.76134652 | 0.957 | 0.928 | 0 |
| RPL8 | 0 | 0.75840714 | 0.933 | 0.87 | 0 |
| SOD1 | 0 | 0.74633443 | 0.752 | 0.575 | 0 |
| PABPC1 | 1.16E-176 | 0.74525103 | 0.791 | 0.716 | 3.04E-172 |
| IER5 | 1.60E-168 | 0.74427733 | 0.453 | 0.303 | 4.18E-164 |
| JUND | 0 | 0.74256141 | 0.891 | 0.794 | 0 |
| RPS9 | 0 | 0.74054184 | 0.935 | 0.869 | 0 |
| RPS11 | 0 | 0.73757032 | 0.936 | 0.872 | 0 |
| RPL7 | 0 | 0.73307401 | 0.911 | 0.818 | 0 |
| RPLP0 | 0 | 0.72981151 | 0.903 | 0.813 | 0 |
| DNAJB4 | 0 | 0.72257914 | 0.413 | 0.197 | 0 |
| RPL3 | 0 | 0.71795812 | 0.937 | 0.89 | 0 |
| RACK1 | 0 | 0.70119778 | 0.915 | 0.831 | 0 |
| RPL21 | 0 | 0.68517829 | 0.934 | 0.879 | 0 |
| RPL10A | 0 | 0.67876443 | 0.921 | 0.853 | 0 |
| GADD45B | 2.03E-178 | 0.67784472 | 0.622 | 0.474 | 5.32E-174 |
| RPLP1 | 0 | 0.67774232 | 0.97 | 0.95 | 0 |
| RPL4 | 0 | 0.67095136 | 0.858 | 0.735 | 0 |
| RPL9 | 0 | 0.66972342 | 0.938 | 0.881 | 0 |
| RPS8 | 0 | 0.66735076 | 0.956 | 0.922 | 0 |
| RPL27A | 0 | 0.66536282 | 0.94 | 0.892 | 0 |
| RPL29 | 0 | 0.6638881 | 0.942 | 0.896 | 0 |
| MT-ND5 | 6.22E-17 | 0.6613932 | 0.822 | 0.892 | 1.63E-12 |
| RPS13 | 0 | 0.65958968 | 0.934 | 0.868 | 0 |
| DNAJA4 | 3.88E-294 | 0.65917001 | 0.3 | 0.125 | 1.02E-289 |
| RPSA | 0 | 0.65828156 | 0.904 | 0.836 | 0 |
| CREM | 0 | 0.65653413 | 0.511 | 0.27 | 0 |
| RORA | 3.32E-119 | 0.65321356 | 0.492 | 0.378 | 8.69E-115 |
| RPL18 | 0 | 0.65314728 | 0.939 | 0.896 | 0 |
| RPL11 | 0 | 0.65230681 | 0.953 | 0.917 | 0 |
| UBA52 | 0 | 0.64547576 | 0.919 | 0.851 | 0 |
| RPL19 | 0 | 0.6405417 | 0.954 | 0.92 | 0 |
| RGS2 | 6.21E-268 | 0.63979465 | 0.617 | 0.419 | 1.62E-263 |
| JUNB | 1.88E-284 | 0.6384363 | 0.802 | 0.616 | 4.92E-280 |
| EEF1A1 | 0 | 0.63356734 | 0.98 | 0.971 | 0 |
| UBB | 7.87E-272 | 0.6331212 | 0.847 | 0.733 | 2.06E-267 |
| RPS4X | 0 | 0.63183994 | 0.944 | 0.905 | 0 |
| RPL10 | 0 | 0.63173954 | 0.966 | 0.948 | 0 |
| RPS4Y1 | 0 | 0.63037186 | 0.434 | 0.196 | 0 |
| RPS5 | 0 | 0.62823727 | 0.902 | 0.824 | 0 |
| RPS3 | 0 | 0.62592077 | 0.945 | 0.903 | 0 |
| RPS12 | 0 | 0.62431694 | 0.956 | 0.92 | 0 |
| SRGN | 1.86E-275 | 0.62318953 | 0.87 | 0.759 | 4.88E-271 |
| RPL23A | 0 | 0.6136309 | 0.95 | 0.904 | 0 |
| RPS14 | 0 | 0.61298702 | 0.947 | 0.911 | 0 |
| EIF1 | 0 | 0.61092122 | 0.947 | 0.88 | 0 |
| RPS19 | 0 | 0.60813384 | 0.958 | 0.924 | 0 |
| RPL5 | 0 | 0.60763406 | 0.922 | 0.862 | 0 |
| SLC2A3 | 5.82E-215 | 0.60714135 | 0.647 | 0.487 | 1.52E-210 |
| RPS20 | 0 | 0.60530973 | 0.938 | 0.876 | 0 |
| PLIN2 | 3.32E-230 | 0.60139687 | 0.313 | 0.153 | 8.68E-226 |
| RPL23 | 1.14E-302 | 0.59401717 | 0.9 | 0.817 | 2.99E-298 |
| EEF1B2 | 2.24E-285 | 0.59371596 | 0.861 | 0.754 | 5.85E-281 |
| DUSP2 | 3.52E-208 | 0.59271637 | 0.656 | 0.486 | 9.20E-204 |
| HSPA5 | 3.24E-159 | 0.59199285 | 0.578 | 0.443 | 8.48E-155 |
| MT-CO2 | 2.73E-221 | 0.59148845 | 0.979 | 0.981 | 7.15E-217 |
| RPS27A | 0 | 0.58365855 | 0.954 | 0.919 | 0 |
| RPS23 | 0 | 0.57302409 | 0.949 | 0.904 | 0 |
| NACA | 0 | 0.57052953 | 0.928 | 0.857 | 0 |
| NAMPT | 9.15E-190 | 0.5688537 | 0.413 | 0.254 | 2.39E-185 |
| RPL7A | 0 | 0.5633449 | 0.941 | 0.898 | 0 |
| ISG15 | 2.73E-201 | 0.55536076 | 0.495 | 0.317 | 7.14E-197 |
| RPL12 | 0 | 0.55531463 | 0.95 | 0.898 | 0 |
| PPIA | 0 | 0.55280206 | 0.876 | 0.769 | 0 |
| DNAJB6 | 1.69E-307 | 0.55246287 | 0.573 | 0.372 | 4.43E-303 |
| RPL28 | 0 | 0.55044642 | 0.965 | 0.95 | 0 |
| EEF2 | 4.56E-266 | 0.54880205 | 0.736 | 0.585 | 1.19E-261 |
| RPS15 | 0 | 0.54787024 | 0.948 | 0.911 | 0 |
| RPL6 | 0 | 0.54728637 | 0.947 | 0.904 | 0 |
| RPL32 | 0 | 0.54195489 | 0.959 | 0.929 | 0 |
| NR4A1 | 1.40E-209 | 0.53109018 | 0.472 | 0.296 | 3.68E-205 |
| CYCS | 3.47E-166 | 0.52692491 | 0.537 | 0.388 | 9.07E-162 |
| RPL26 | 0 | 0.52443908 | 0.954 | 0.91 | 0 |
| NPM1 | 8.89E-288 | 0.523491 | 0.829 | 0.692 | 2.33E-283 |
| RPL24 | 0 | 0.51803401 | 0.935 | 0.871 | 0 |
| RPL27 | 3.36E-294 | 0.51650465 | 0.901 | 0.822 | 8.80E-290 |
| BIRC3 | 1.93E-124 | 0.51585135 | 0.601 | 0.482 | 5.04E-120 |
| RPL14 | 0 | 0.51226698 | 0.95 | 0.909 | 0 |
| HLA-B | 0 | 0.50547247 | 0.97 | 0.945 | 0 |
| RPS25 | 0 | 0.49976595 | 0.952 | 0.907 | 0 |
| TMSB4X | 4.67E-258 | 0.49559978 | 0.985 | 0.976 | 1.22E-253 |
| CLK1 | 9.57E-246 | 0.49494939 | 0.618 | 0.442 | 2.50E-241 |
| BCAS2 | 5.60E-217 | 0.4941198 | 0.399 | 0.23 | 1.46E-212 |
| GPBP1 | 5.91E-240 | 0.49370376 | 0.728 | 0.593 | 1.55E-235 |
| ACTG1 | 8.86E-142 | 0.49319811 | 0.903 | 0.849 | 2.32E-137 |
| OAZ1 | 1.41E-270 | 0.4900347 | 0.842 | 0.709 | 3.70E-266 |
| HINT1 | 1.07E-249 | 0.4892818 | 0.755 | 0.607 | 2.81E-245 |
| NEAT1 | 1.50E-48 | 0.48877677 | 0.789 | 0.773 | 3.92E-44 |
| CNOT6L | 1.31E-129 | 0.4881846 | 0.546 | 0.429 | 3.44E-125 |
| CYTIP | 3.13E-112 | 0.48788345 | 0.605 | 0.505 | 8.19E-108 |
| BTG2 | 1.87E-152 | 0.48534933 | 0.688 | 0.564 | 4.89E-148 |
| LTB | 8.35E-178 | 0.48520116 | 0.657 | 0.498 | 2.18E-173 |
| RPLP2 | 1.29E-249 | 0.48113715 | 0.956 | 0.921 | 3.37E-245 |
| EIF3E | 8.37E-271 | 0.47103847 | 0.632 | 0.445 | 2.19E-266 |
| EEF1D | 7.54E-283 | 0.46889834 | 0.874 | 0.767 | 1.97E-278 |
| LDHA | 1.04E-170 | 0.46838751 | 0.634 | 0.492 | 2.71E-166 |
| RGS1 | 5.24E-40 | 0.46814049 | 0.576 | 0.524 | 1.37E-35 |
| TSPYL2 | 6.62E-144 | 0.46570544 | 0.574 | 0.44 | 1.73E-139 |
| COX4I1 | 3.65E-261 | 0.46526571 | 0.801 | 0.67 | 9.55E-257 |
| TNFAIP3 | 2.24E-79 | 0.46227553 | 0.535 | 0.434 | 5.87E-75 |
| RPS7 | 3.98E-262 | 0.46101674 | 0.944 | 0.901 | 1.04E-257 |
| SLC25A6 | 2.51E-248 | 0.45954927 | 0.687 | 0.52 | 6.55E-244 |
| BTF3 | 4.25E-241 | 0.45938746 | 0.83 | 0.709 | 1.11E-236 |
| LDHB | 9.85E-246 | 0.4583765 | 0.617 | 0.433 | 2.58E-241 |
| SATB1 | 2.18E-199 | 0.45643173 | 0.257 | 0.118 | 5.70E-195 |
| PTMA | 2.35E-279 | 0.45387079 | 0.975 | 0.949 | 6.14E-275 |
| NAP1L1 | 3.53E-167 | 0.4505571 | 0.723 | 0.609 | 9.25E-163 |
| CD69 | 5.97E-45 | 0.44666905 | 0.683 | 0.634 | 1.56E-40 |
| RPL30 | 9.04E-261 | 0.44660369 | 0.955 | 0.923 | 2.36E-256 |
| CD7 | 7.00E-126 | 0.44514525 | 0.573 | 0.444 | 1.83E-121 |
| NR4A2 | 5.23E-129 | 0.43927037 | 0.419 | 0.277 | 1.37E-124 |
| ID2 | 6.79E-41 | 0.43863877 | 0.497 | 0.432 | 1.78E-36 |
| RPS15A | 1.26E-260 | 0.43776811 | 0.958 | 0.92 | 3.28E-256 |
| PNRC1 | 1.30E-114 | 0.43737028 | 0.805 | 0.751 | 3.39E-110 |
| RPL31 | 9.32E-198 | 0.43406249 | 0.897 | 0.82 | 2.44E-193 |
| GAPDH | 2.45E-108 | 0.43336955 | 0.865 | 0.795 | 6.42E-104 |
| AHSA1 | 6.29E-261 | 0.43295861 | 0.437 | 0.248 | 1.65E-256 |
| CHORDC1 | 7.87E-149 | 0.4324815 | 0.494 | 0.357 | 2.06E-144 |
| FKBP4 | 7.63E-246 | 0.43126011 | 0.261 | 0.108 | 2.00E-241 |
| SNHG29 | 8.62E-235 | 0.43029537 | 0.671 | 0.497 | 2.26E-230 |
| KLF6 | 3.22E-45 | 0.42424295 | 0.74 | 0.711 | 8.43E-41 |
| SOCS3 | 1.22E-254 | 0.42292897 | 0.356 | 0.175 | 3.19E-250 |
| ZFAS1 | 5.98E-219 | 0.4220521 | 0.717 | 0.564 | 1.57E-214 |
| LGALS3 | 2.28E-125 | 0.4210656 | 0.375 | 0.248 | 5.96E-121 |
| KLF2 | 8.85E-68 | 0.41959793 | 0.468 | 0.373 | 2.31E-63 |
| EIF3F | 1.80E-249 | 0.41906978 | 0.622 | 0.438 | 4.71E-245 |
| DUSP5 | 1.90E-230 | 0.41881694 | 0.291 | 0.131 | 4.97E-226 |
| H3-3A | 1.63E-206 | 0.41782826 | 0.877 | 0.777 | 4.27E-202 |
| ISG20 | 8.02E-152 | 0.41775 | 0.609 | 0.469 | 2.10E-147 |
| RPL22 | 7.85E-202 | 0.41112728 | 0.897 | 0.811 | 2.06E-197 |
| FAU | 3.24E-246 | 0.40774073 | 0.938 | 0.892 | 8.48E-242 |
| RPL35 | 5.78E-207 | 0.40767933 | 0.925 | 0.872 | 1.51E-202 |
| UBE2S | 3.88E-211 | 0.40567582 | 0.366 | 0.203 | 1.01E-206 |
| FXYD5 | 6.87E-203 | 0.40410952 | 0.756 | 0.617 | 1.80E-198 |
| PFDN5 | 4.57E-227 | 0.40406161 | 0.867 | 0.768 | 1.20E-222 |
| HNRNPA1 | 9.90E-161 | 0.40268243 | 0.836 | 0.741 | 2.59E-156 |
| SLC25A5 | 1.03E-203 | 0.3988131 | 0.48 | 0.308 | 2.69E-199 |
| RPS3A | 1.01E-170 | 0.39709355 | 0.943 | 0.902 | 2.64E-166 |
| BTG1 | 1.93E-39 | 0.39606692 | 0.94 | 0.927 | 5.05E-35 |
| SARAF | 1.10E-136 | 0.39168755 | 0.768 | 0.667 | 2.87E-132 |
| ZNF331 | 9.48E-75 | 0.38835647 | 0.334 | 0.236 | 2.48E-70 |
| GNAS | 7.73E-187 | 0.38411616 | 0.767 | 0.639 | 2.02E-182 |
| RPL34 | 5.57E-185 | 0.3839537 | 0.963 | 0.934 | 1.46E-180 |
| CLEC2B | 7.16E-85 | 0.37670035 | 0.475 | 0.373 | 1.87E-80 |
| PMAIP1 | 5.40E-131 | 0.37459877 | 0.344 | 0.211 | 1.41E-126 |
| ATP5MC2 | 1.37E-189 | 0.3743252 | 0.745 | 0.599 | 3.58E-185 |
| CDKN1A | 1.20E-278 | 0.37400013 | 0.292 | 0.119 | 3.13E-274 |
| EIF3H | 6.54E-206 | 0.37280308 | 0.618 | 0.452 | 1.71E-201 |
| TNFRSF4 | 1.87E-89 | 0.37237521 | 0.271 | 0.169 | 4.90E-85 |
| REL | 1.52E-81 | 0.37038005 | 0.497 | 0.394 | 3.96E-77 |
| FOS | 1.76E-48 | 0.36821259 | 0.655 | 0.575 | 4.61E-44 |
| LGALS1 | 7.10E-38 | 0.367132 | 0.499 | 0.428 | 1.86E-33 |
| YPEL5 | 5.76E-137 | 0.36553564 | 0.472 | 0.335 | 1.51E-132 |
| EIF3K | 1.30E-183 | 0.36491242 | 0.699 | 0.554 | 3.40E-179 |
| YBX1 | 6.46E-126 | 0.36477116 | 0.738 | 0.626 | 1.69E-121 |
| PPP1R2 | 4.72E-119 | 0.36461645 | 0.603 | 0.483 | 1.23E-114 |
| ENO1 | 1.21E-75 | 0.36366003 | 0.528 | 0.431 | 3.17E-71 |
| SSR4 | 2.52E-145 | 0.36203994 | 0.678 | 0.546 | 6.60E-141 |
| TNFSF14 | 1.57E-65 | 0.36196635 | 0.267 | 0.188 | 4.10E-61 |
| RPL39 | 3.50E-161 | 0.3605808 | 0.95 | 0.912 | 9.15E-157 |
| ZFP36 | 8.41E-63 | 0.35986195 | 0.608 | 0.517 | 2.20E-58 |
| TUBA1B | 5.94E-152 | 0.35872723 | 0.518 | 0.369 | 1.55E-147 |
| SELENOK | 3.03E-148 | 0.35686639 | 0.53 | 0.379 | 7.93E-144 |
| PIM1 | 3.30E-151 | 0.35452493 | 0.33 | 0.194 | 8.63E-147 |
| SMCHD1 | 2.16E-52 | 0.35337002 | 0.647 | 0.601 | 5.64E-48 |
| CD48 | 4.64E-160 | 0.3513235 | 0.757 | 0.642 | 1.21E-155 |
| HERPUD1 | 1.31E-119 | 0.350322 | 0.485 | 0.356 | 3.43E-115 |
| PDE4D | 7.18E-112 | 0.34992845 | 0.3 | 0.186 | 1.88E-107 |
| MT2A | 4.69E-09 | 0.34950061 | 0.431 | 0.411 | 0.00012282 |
| ARPC3 | 4.70E-137 | 0.34885764 | 0.721 | 0.604 | 1.23E-132 |
| GAS5 | 7.59E-144 | 0.34554644 | 0.695 | 0.569 | 1.99E-139 |
| FOSL2 | 1.22E-109 | 0.3442948 | 0.278 | 0.166 | 3.20E-105 |
| TNFRSF18 | 3.19E-102 | 0.34406775 | 0.29 | 0.178 | 8.33E-98 |
| PTGES3 | 3.24E-85 | 0.34078909 | 0.65 | 0.556 | 8.48E-81 |
| ARPC2 | 1.41E-127 | 0.33973001 | 0.797 | 0.703 | 3.68E-123 |
| EZR | 2.02E-47 | 0.339096 | 0.614 | 0.553 | 5.28E-43 |
| NFKBIZ | 1.31E-147 | 0.33753944 | 0.279 | 0.152 | 3.43E-143 |
| RPL36 | 1.10E-145 | 0.33753848 | 0.934 | 0.88 | 2.88E-141 |
| CHCHD2 | 7.30E-119 | 0.33392195 | 0.687 | 0.572 | 1.91E-114 |
| TCP1 | 9.46E-147 | 0.33237017 | 0.41 | 0.268 | 2.48E-142 |
| RPL35A | 2.39E-151 | 0.33143858 | 0.939 | 0.894 | 6.25E-147 |
| DEDD2 | 6.90E-178 | 0.33078635 | 0.356 | 0.204 | 1.80E-173 |
| SESN3 | 4.99E-112 | 0.33073123 | 0.259 | 0.151 | 1.31E-107 |
| SNHG8 | 5.92E-158 | 0.32965308 | 0.517 | 0.36 | 1.55E-153 |
| CD55 | 1.48E-186 | 0.32951497 | 0.307 | 0.158 | 3.87E-182 |
| G3BP2 | 2.62E-73 | 0.32822149 | 0.498 | 0.404 | 6.85E-69 |
| MXD1 | 2.51E-199 | 0.32661181 | 0.253 | 0.113 | 6.56E-195 |
| LINC-PINT | 7.08E-92 | 0.32651347 | 0.576 | 0.481 | 1.85E-87 |
| UBALD2 | 3.55E-168 | 0.32624122 | 0.351 | 0.206 | 9.28E-164 |
| MT-ND2 | 4.81E-29 | 0.32570505 | 0.935 | 0.946 | 1.26E-24 |
| MT-ATP6 | 6.26E-114 | 0.32540816 | 0.993 | 0.995 | 1.64E-109 |
| NOP53 | 3.13E-137 | 0.32521001 | 0.733 | 0.626 | 8.20E-133 |
| CGAS | 2.03E-134 | 0.3236889 | 0.272 | 0.153 | 5.31E-130 |
| HNRNPC | 5.05E-97 | 0.32133521 | 0.712 | 0.627 | 1.32E-92 |
| RPL41 | 4.10E-174 | 0.31967077 | 0.964 | 0.937 | 1.07E-169 |
| RPL37A | 1.38E-118 | 0.31866453 | 0.934 | 0.888 | 3.62E-114 |
| RPL36AL | 1.41E-150 | 0.31844468 | 0.794 | 0.67 | 3.69E-146 |
| COTL1 | 1.90E-92 | 0.31762883 | 0.641 | 0.531 | 4.96E-88 |
| SERP1 | 1.62E-137 | 0.31545224 | 0.588 | 0.451 | 4.24E-133 |
| CXCR4 | 7.60E-48 | 0.31538376 | 0.777 | 0.741 | 1.99E-43 |
| YWHAB | 1.63E-108 | 0.31446544 | 0.782 | 0.698 | 4.26E-104 |
| RSRP1 | 3.13E-101 | 0.3102839 | 0.722 | 0.647 | 8.19E-97 |
| TNFAIP8 | 1.03E-68 | 0.30963081 | 0.528 | 0.44 | 2.69E-64 |
| ATF4 | 9.04E-156 | 0.3089133 | 0.372 | 0.229 | 2.36E-151 |
| ARID5B | 2.24E-79 | 0.30779655 | 0.531 | 0.425 | 5.85E-75 |
| SUB1 | 1.36E-127 | 0.30623862 | 0.708 | 0.587 | 3.55E-123 |
| MYL12A | 6.37E-84 | 0.30523174 | 0.822 | 0.761 | 1.67E-79 |
| STIP1 | 5.62E-144 | 0.30303153 | 0.325 | 0.192 | 1.47E-139 |
| IL7R | 4.06E-59 | 0.3026166 | 0.657 | 0.573 | 1.06E-54 |
| CCNI | 6.00E-123 | 0.30083411 | 0.665 | 0.546 | 1.57E-118 |
| ANKRD12 | 6.84E-34 | 0.29905064 | 0.7 | 0.667 | 1.79E-29 |
| MT-CO3 | 4.40E-28 | 0.2972537 | 0.976 | 0.989 | 1.15E-23 |
| NDUFS5 | 6.57E-124 | 0.29722817 | 0.59 | 0.459 | 1.72E-119 |
| RNASET2 | 7.66E-132 | 0.29694379 | 0.409 | 0.273 | 2.01E-127 |
| TPI1 | 2.13E-82 | 0.29662717 | 0.529 | 0.419 | 5.58E-78 |
| RPS17 | 4.66E-57 | 0.29557329 | 0.61 | 0.539 | 1.22E-52 |
| ERN1 | 2.14E-83 | 0.29347815 | 0.307 | 0.206 | 5.61E-79 |
| RPL22L1 | 9.06E-103 | 0.29169058 | 0.383 | 0.262 | 2.37E-98 |
| DOK2 | 5.17E-81 | 0.29103708 | 0.417 | 0.317 | 1.35E-76 |
| UQCRB | 2.20E-135 | 0.2898693 | 0.775 | 0.649 | 5.75E-131 |
| RAN | 1.11E-101 | 0.28803575 | 0.587 | 0.47 | 2.91E-97 |
| KDM6B | 7.76E-94 | 0.28688874 | 0.269 | 0.17 | 2.03E-89 |
| RPL37 | 7.54E-109 | 0.28516978 | 0.964 | 0.934 | 1.97E-104 |
| SDCBP | 2.12E-111 | 0.28422046 | 0.416 | 0.291 | 5.54E-107 |
| H2AZ1 | 1.45E-95 | 0.28403812 | 0.534 | 0.42 | 3.78E-91 |
| B2M | 1.48E-144 | 0.28312383 | 0.993 | 0.989 | 3.87E-140 |
| RPS24 | 5.10E-119 | 0.28283602 | 0.959 | 0.933 | 1.33E-114 |
| HIF1A | 2.67E-57 | 0.28259748 | 0.378 | 0.297 | 6.97E-53 |
| UBE2B | 1.52E-122 | 0.28227998 | 0.511 | 0.374 | 3.98E-118 |
| MALT1 | 5.78E-57 | 0.28214899 | 0.299 | 0.219 | 1.51E-52 |
| ANP32B | 1.07E-106 | 0.28125048 | 0.592 | 0.473 | 2.79E-102 |
| SEC61B | 1.16E-79 | 0.28116226 | 0.463 | 0.357 | 3.04E-75 |
| CHD1 | 4.69E-62 | 0.28059602 | 0.422 | 0.34 | 1.23E-57 |
| PCBP1 | 6.76E-125 | 0.28006259 | 0.52 | 0.385 | 1.77E-120 |
| SELL | 3.90E-91 | 0.2794543 | 0.348 | 0.238 | 1.02E-86 |
| BATF | 2.79E-93 | 0.27872951 | 0.343 | 0.229 | 7.30E-89 |
| RHOH | 7.63E-75 | 0.27817629 | 0.465 | 0.367 | 2.00E-70 |
| EIF4A2 | 1.47E-108 | 0.27816285 | 0.753 | 0.656 | 3.86E-104 |
| HSP90B1 | 3.52E-44 | 0.27711233 | 0.566 | 0.498 | 9.22E-40 |
| PRRC2C | 1.23E-48 | 0.27669038 | 0.759 | 0.736 | 3.21E-44 |
| BTG3 | 1.67E-115 | 0.27641445 | 0.321 | 0.2 | 4.38E-111 |
| MZT2A | 4.81E-142 | 0.2763288 | 0.467 | 0.319 | 1.26E-137 |
| TUBB4B | 5.26E-144 | 0.27525318 | 0.409 | 0.261 | 1.38E-139 |
| MRPL18 | 1.77E-138 | 0.27501244 | 0.265 | 0.145 | 4.63E-134 |
| SMAP2 | 6.55E-62 | 0.27444901 | 0.526 | 0.438 | 1.71E-57 |
| DYNLL1 | 6.53E-69 | 0.27381739 | 0.578 | 0.494 | 1.71E-64 |
| CIB1 | 8.37E-114 | 0.27248876 | 0.549 | 0.422 | 2.19E-109 |
| BAZ1A | 6.95E-87 | 0.2721304 | 0.421 | 0.316 | 1.82E-82 |
| LAPTM5 | 9.22E-104 | 0.27073175 | 0.689 | 0.581 | 2.41E-99 |
| PIM3 | 9.43E-99 | 0.2700239 | 0.363 | 0.246 | 2.47E-94 |
| SUMO2 | 3.93E-109 | 0.26983501 | 0.694 | 0.574 | 1.03E-104 |
| PTPRC | 1.97E-60 | 0.26978591 | 0.957 | 0.957 | 5.15E-56 |
| CCT4 | 6.57E-106 | 0.26927898 | 0.412 | 0.291 | 1.72E-101 |
| CFL1 | 1.92E-69 | 0.26911305 | 0.864 | 0.803 | 5.03E-65 |
| MYL6 | 5.32E-57 | 0.2686924 | 0.849 | 0.783 | 1.39E-52 |
| RHOG | 1.33E-124 | 0.26628689 | 0.427 | 0.292 | 3.49E-120 |
| SERPINB9 | 1.84E-74 | 0.26590829 | 0.305 | 0.209 | 4.83E-70 |
| CDV3 | 1.41E-65 | 0.26574868 | 0.529 | 0.441 | 3.69E-61 |
| SNHG16 | 1.33E-137 | 0.26485648 | 0.302 | 0.174 | 3.49E-133 |
| SNU13 | 6.67E-116 | 0.26484604 | 0.544 | 0.411 | 1.74E-111 |
| DDX21 | 2.36E-82 | 0.2647567 | 0.42 | 0.313 | 6.16E-78 |
| PRNP | 1.85E-91 | 0.26462215 | 0.322 | 0.216 | 4.83E-87 |
| STAT3 | 3.45E-74 | 0.26453921 | 0.57 | 0.478 | 9.03E-70 |
| GBP5 | 7.40E-42 | 0.26346372 | 0.441 | 0.368 | 1.94E-37 |
| PPIB | 3.24E-55 | 0.26336587 | 0.597 | 0.515 | 8.48E-51 |
| PFN1 | 1.74E-49 | 0.26333686 | 0.87 | 0.831 | 4.56E-45 |
| GPR183 | 5.25E-31 | 0.26297742 | 0.473 | 0.419 | 1.37E-26 |
| SERF2 | 2.77E-96 | 0.26256444 | 0.882 | 0.806 | 7.24E-92 |
| SERTAD1 | 1.19E-181 | 0.2621552 | 0.255 | 0.12 | 3.11E-177 |
| CCR7 | 5.32E-61 | 0.2618837 | 0.263 | 0.184 | 1.39E-56 |
| SPOCK2 | 4.85E-32 | 0.26141061 | 0.576 | 0.53 | 1.27E-27 |
| SSR2 | 3.12E-104 | 0.26087009 | 0.59 | 0.471 | 8.16E-100 |
| APRT | 2.48E-90 | 0.25944487 | 0.556 | 0.447 | 6.48E-86 |
| RBM39 | 2.66E-65 | 0.2589162 | 0.789 | 0.748 | 6.96E-61 |
| EDF1 | 5.12E-107 | 0.25856578 | 0.649 | 0.534 | 1.34E-102 |
| DDX3X | 1.89E-25 | 0.25830134 | 0.647 | 0.622 | 4.95E-21 |
| FAM107B | 5.09E-41 | 0.25792034 | 0.619 | 0.559 | 1.33E-36 |
| SYTL3 | 2.21E-66 | 0.25721921 | 0.389 | 0.296 | 5.77E-62 |
| CSRNP1 | 1.73E-170 | 0.2570446 | 0.273 | 0.136 | 4.53E-166 |
| UXT | 1.52E-117 | 0.25682852 | 0.497 | 0.365 | 3.98E-113 |
| SOD2 | 5.47E-80 | 0.25630248 | 0.329 | 0.231 | 1.43E-75 |
| SAP18 | 3.61E-89 | 0.25351365 | 0.544 | 0.433 | 9.45E-85 |
| FOXP1 | 1.13E-18 | 0.25318294 | 0.473 | 0.444 | 2.96E-14 |
| MZT2B | 2.22E-106 | 0.25303306 | 0.583 | 0.458 | 5.80E-102 |
| PSMB4 | 4.45E-149 | 0.25167333 | 0.312 | 0.177 | 1.16E-144 |
| EIF5 | 1.29E-94 | 0.25162388 | 0.49 | 0.377 | 3.37E-90 |
| RAB11FIP1 | 7.62E-70 | 0.2504453 | 0.329 | 0.236 | 1.99E-65 |
| HNRNPU | 6.21E-27 | 0.25014224 | 0.794 | 0.782 | 1.62E-22 |
| CD2 | 5.76E-71 | -0.26114002 | 0.754 | 0.813 | 1.51E-66 |
| CD8A | 5.18E-66 | -0.26835311 | 0.239 | 0.338 | 1.36E-61 |
| HCST | 2.81E-63 | -0.27164128 | 0.642 | 0.701 | 7.35E-59 |
| AHNAK | 3.34E-173 | -0.38532839 | 0.549 | 0.702 | 8.74E-169 |
| CCL5 | 1.93E-109 | -0.40896897 | 0.43 | 0.581 | 5.06E-105 |
| GZMK | 1.49E-100 | -0.4639463 | 0.3 | 0.429 | 3.90E-96 |
| KRT14 | 0 | -0.50664156 | 0.011 | 0.293 | 0 |
| CRIP1 | 1.60E-205 | -0.56072497 | 0.397 | 0.566 | 4.19E-201 |
| CCL4L2 | 2.70E-282 | -1.05554618 | 0.114 | 0.314 | 7.08E-278 |

**Supplementary File 1b. List of genes with statistically significant differences between CD8^+^ Trm subgroups in EOLP and NEOLP.**

|  | p_val | | avg_log2FC | | pct.1 | | pct.2 | p_val_adj | |
| --- | --- | --- | --- | --- | --- | --- | --- | --- | --- |
| NFKBIA | 1.56E-73 | | 2.16884882 | | 0.757 | | 0.529 | 4.09E-69 | |
| GZMB | 0.01504761 | | 1.91436612 | | 0.522 | | 0.553 | 1 | |
| GNLY | 6.76E-26 | | 1.9073165 | | 0.369 | | 0.205 | 1.77E-21 | |
| IFNG | 1.95E-06 | | 1.75119787 | | 0.425 | | 0.371 | 0.05097991 | |
| HSP90AB1 | 1.90E-80 | | 1.45178708 | | 0.94 | | 0.88 | 4.96E-76 | |
| FTH1 | 3.86E-71 | | 1.40536183 | | 0.975 | | 0.949 | 1.01E-66 | |
| DDIT4 | 8.52E-59 | | 1.31030899 | | 0.757 | | 0.51 | 2.23E-54 | |
| HSPD1 | 3.23E-73 | | 1.30308386 | | 0.855 | | 0.646 | 8.46E-69 | |
| SERPINH1 | 1.45E-49 | | 1.30068918 | | 0.461 | | 0.217 | 3.78E-45 | |
| ZFAND2A | 2.54E-61 | | 1.29284149 | | 0.502 | | 0.226 | 6.65E-57 | |
| HSP90AA1 | 4.92E-85 | | 1.28167891 | | 0.992 | | 0.957 | 1.29E-80 | |
| HSPA6 | 3.53E-58 | | 1.26793302 | | 0.713 | | 0.442 | 9.23E-54 | |
| DNAJA1 | 4.31E-73 | | 1.2442147 | | 0.836 | | 0.631 | 1.13E-68 | |
| FTL | 7.54E-89 | | 1.17602863 | | 0.959 | | 0.884 | 1.97E-84 | |
| HSPH1 | 1.08E-54 | | 1.10028573 | | 0.804 | | 0.564 | 2.83E-50 | |
| MT2A | 0.0001701 | | 1.06099366 | | 0.494 | | 0.473 | 1 | |
| HSPA1A | 4.27E-65 | | 1.03570985 | | 0.922 | | 0.795 | 1.12E-60 | |
| RPS16 | 9.50E-98 | | 1.02362172 | | 0.988 | | 0.979 | 2.49E-93 | |
| MT-ND5 | 4.06E-08 | | 1.01576691 | | 0.844 | | 0.918 | 0.00106276 | |
| HSPA5 | 3.76E-42 | | 1.0099468 | | 0.67 | | 0.488 | 9.85E-38 | |
| PPP1R15A | 8.70E-77 | | 0.99331187 | | 0.896 | | 0.646 | 2.28E-72 | |
| BAG3 | 5.38E-69 | | 0.99126908 | | 0.484 | | 0.181 | 1.41E-64 | |
| RPLP0 | 1.14E-62 | | 0.97759563 | | 0.955 | | 0.912 | 2.99E-58 | |
| CACYBP | 6.27E-63 | | 0.96021256 | | 0.78 | | 0.548 | 1.64E-58 | |
| RPS2 | 1.72E-96 | | 0.95603092 | | 0.993 | | 0.992 | 4.50E-92 | |
| PABPC1 | 8.03E-29 | | 0.95471768 | | 0.821 | | 0.754 | 2.10E-24 | |
| CREM | 3.49E-82 | | 0.94035727 | | 0.582 | | 0.246 | 9.13E-78 | |
| HSPA8 | 1.88E-61 | | 0.93305843 | | 0.951 | | 0.856 | 4.91E-57 | |
| RORA | 2.13E-28 | | 0.92285052 | | 0.594 | | 0.45 | 5.58E-24 | |
| LDHA | 1.89E-29 | | 0.89172022 | | 0.69 | | 0.573 | 4.93E-25 | |
| ENO1 | 1.11E-15 | | 0.8843912 | | 0.599 | | 0.515 | 2.90E-11 | |
| SAT1 | 5.70E-46 | | 0.88064244 | | 0.799 | | 0.598 | 1.49E-41 | |
| RPL15 | 6.07E-76 | | 0.86829812 | | 0.986 | | 0.968 | 1.59E-71 | |
| TPT1 | 1.52E-110 | | 0.86755124 | | 0.998 | | 0.993 | 3.98E-106 | |
| SRGN | 6.65E-59 | | 0.84547452 | | 0.953 | | 0.894 | 1.74E-54 | |
| TNFAIP3 | 5.14E-28 | | 0.84494133 | | 0.675 | | 0.554 | 1.34E-23 | |
| NEAT1 | 4.89E-12 | | 0.83992204 | | 0.803 | | 0.793 | 1.28E-07 | |
| JUND | 9.12E-52 | | 0.83476683 | | 0.929 | | 0.881 | 2.39E-47 | |
| DUSP2 | 9.15E-50 | | 0.82160117 | | 0.778 | | 0.554 | 2.39E-45 | |
| RPL18A | 1.48E-91 | | 0.817689 | | 0.989 | | 0.988 | 3.86E-87 | |
| IER5 | 3.26E-34 | | 0.81644446 | | 0.585 | | 0.374 | 8.53E-30 | |
| RPL8 | 6.67E-75 | | 0.81011835 | | 0.986 | | 0.972 | 1.75E-70 | |
| LGALS1 | 1.27E-15 | | 0.80858047 | | 0.707 | | 0.629 | 3.32E-11 | |
| GAPDH | 7.82E-17 | | 0.80722373 | | 0.937 | | 0.909 | 2.05E-12 | |
| RPL13A | 4.59E-72 | | 0.80009002 | | 0.993 | | 0.99 | 1.20E-67 | |
| GADD45B | 1.11E-41 | | 0.79775276 | | 0.762 | | 0.544 | 2.89E-37 | |
| RPS9 | 2.45E-92 | | 0.79398621 | | 0.987 | | 0.966 | 6.41E-88 | |
| RPL3 | 5.48E-76 | | 0.77973667 | | 0.985 | | 0.966 | 1.43E-71 | |
| LTB | 3.22E-40 | | 0.77259976 | | 0.613 | | 0.375 | 8.42E-36 | |
| RPL13 | 6.38E-87 | | 0.77203352 | | 0.995 | | 0.999 | 1.67E-82 | |
| KLRB1 | 1.79E-15 | | 0.76726859 | | 0.386 | | 0.263 | 4.69E-11 | |
| DNAJA4 | 3.26E-27 | | 0.76603745 | | 0.404 | | 0.232 | 8.52E-23 | |
| RPS6 | 9.53E-69 | | 0.7624376 | | 0.995 | | 0.984 | 2.49E-64 | |
| HSPB1 | 1.13E-23 | | 0.76221294 | | 0.804 | | 0.714 | 2.95E-19 | |
| PLIN2 | 2.79E-32 | | 0.76189619 | | 0.385 | | 0.188 | 7.30E-28 | |
| RPS11 | 7.90E-71 | | 0.75291511 | | 0.989 | | 0.951 | 2.07E-66 | |
| LGALS3 | 8.70E-25 | | 0.74817733 | | 0.53 | | 0.367 | 2.28E-20 | |
| DNAJB1 | 5.14E-52 | | 0.74625813 | | 0.932 | | 0.758 | 1.34E-47 | |
| MT-CO2 | 2.37E-31 | | 0.74312511 | | 0.983 | | 0.991 | 6.20E-27 | |
| EEF2 | 1.18E-35 | | 0.74198201 | | 0.746 | | 0.604 | 3.08E-31 | |
| RPSA | 7.15E-47 | | 0.74038303 | | 0.959 | | 0.926 | 1.87E-42 | |
| RACK1 | 3.95E-57 | | 0.7402466 | | 0.97 | | 0.935 | 1.03E-52 | |
| RPL7 | 1.71E-56 | | 0.73080298 | | 0.961 | | 0.918 | 4.48E-52 | |
| RPS18 | 3.89E-78 | | 0.72225157 | | 0.996 | | 0.993 | 1.02E-73 | |
| SATB1 | 5.26E-44 | | 0.72179685 | | 0.296 | | 0.095 | 1.38E-39 | |
| RPS20 | 2.51E-68 | | 0.72016861 | | 0.976 | | 0.955 | 6.56E-64 | |
| NPM1 | 1.34E-34 | | 0.71820099 | | 0.861 | | 0.785 | 3.50E-30 | |
| PPIA | 2.56E-37 | | 0.71677268 | | 0.928 | | 0.875 | 6.71E-33 | |
| RPL4 | 7.90E-49 | | 0.70622118 | | 0.899 | | 0.806 | 2.07E-44 | |
| HSPE1 | 1.13E-32 | | 0.70505838 | | 0.875 | | 0.756 | 2.96E-28 | |
| IER3 | 3.14E-25 | | 0.69917843 | | 0.328 | | 0.163 | 8.22E-21 | |
| NAMPT | 4.25E-36 | | 0.69430107 | | 0.467 | | 0.265 | 1.11E-31 | |
| BIRC3 | 1.29E-19 | | 0.69245573 | | 0.604 | | 0.469 | 3.38E-15 | |
| RPL9 | 4.49E-66 | | 0.69069927 | | 0.984 | | 0.971 | 1.17E-61 | |
| RPL27A | 5.17E-70 | | 0.68568961 | | 0.986 | | 0.968 | 1.35E-65 | |
| ACTG1 | 4.53E-23 | | 0.6854395 | | 0.957 | | 0.937 | 1.18E-18 | |
| EEF1A1 | 4.01E-58 | | 0.68512763 | | 0.997 | | 0.999 | 1.05E-53 | |
| JUN | 1.56E-19 | | 0.68252941 | | 0.881 | | 0.817 | 4.09E-15 | |
| RPS4X | 9.13E-44 | | 0.6806936 | | 0.987 | | 0.991 | 2.39E-39 | |
| BHLHE40 | 1.64E-12 | | 0.68057915 | | 0.395 | | 0.292 | 4.30E-08 | |
| RPL5 | 3.52E-43 | | 0.67486932 | | 0.974 | | 0.951 | 9.21E-39 | |
| CYCS | 5.55E-32 | | 0.67065111 | | 0.617 | | 0.426 | 1.45E-27 | |
| RPS5 | 2.46E-49 | | 0.66966723 | | 0.964 | | 0.943 | 6.43E-45 | |
| DUSP5 | 1.02E-48 | | 0.66963891 | | 0.419 | | 0.173 | 2.67E-44 | |
| RPS12 | 1.37E-58 | | 0.66759161 | | 0.993 | | 0.993 | 3.57E-54 | |
| RPL10 | 5.79E-70 | | 0.66552747 | | 0.997 | | 0.999 | 1.52E-65 | |
| RPL23 | 3.54E-32 | | 0.66000678 | | 0.948 | | 0.909 | 9.27E-28 | |
| CYTIP | 1.33E-20 | | 0.65944707 | | 0.675 | | 0.583 | 3.49E-16 | |
| EIF1 | 2.97E-56 | | 0.65609908 | | 0.983 | | 0.966 | 7.77E-52 | |
| DNAJB6 | 1.81E-46 | | 0.65597246 | | 0.681 | | 0.478 | 4.74E-42 | |
| SOD1 | 4.04E-24 | | 0.64870813 | | 0.796 | | 0.71 | 1.06E-19 | |
| CD69 | 5.50E-15 | | 0.64631705 | | 0.817 | | 0.745 | 1.44E-10 | |
| FOSL2 | 7.09E-41 | | 0.64588468 | | 0.342 | | 0.135 | 1.85E-36 | |
| RPLP1 | 1.94E-61 | | 0.64554189 | | 0.999 | | 0.995 | 5.08E-57 | |
| RPS8 | 7.19E-51 | | 0.6419162 | | 0.993 | | 0.989 | 1.88E-46 | |
| RPL18 | 6.32E-52 | | 0.64103543 | | 0.99 | | 0.986 | 1.65E-47 | |
| HLA-B | 3.58E-64 | | 0.63900991 | | 0.994 | | 0.996 | 9.36E-60 | |
| PMAIP1 | 9.13E-32 | | 0.63668466 | | 0.414 | | 0.221 | 2.39E-27 | |
| AQP3 | 2.34E-30 | | 0.63323154 | | 0.32 | | 0.148 | 6.13E-26 | |
| RPS3 | 4.26E-59 | | 0.6318152 | | 0.993 | | 0.986 | 1.11E-54 | |
| HSPA1B | 2.74E-41 | | 0.63010933 | | 0.904 | | 0.797 | 7.16E-37 | |
| RPL29 | 7.65E-61 | | 0.63002313 | | 0.992 | | 0.982 | 2.00E-56 | |
| ID2 | 2.22E-09 | | 0.62586521 | | 0.751 | | 0.714 | 5.80E-05 | |
| RPS13 | 1.82E-54 | | 0.62579229 | | 0.987 | | 0.966 | 4.75E-50 | |
| SSR4 | 1.43E-27 | | 0.62458698 | | 0.786 | | 0.671 | 3.73E-23 | |
| RPL28 | 1.05E-56 | | 0.62160232 | | 0.999 | | 0.999 | 2.74E-52 | |
| UBC | 8.50E-52 | | 0.61807396 | | 0.986 | | 0.953 | 2.22E-47 | |
| NFKBIZ | 9.88E-40 | | 0.61733533 | | 0.402 | | 0.187 | 2.59E-35 | |
| TUBA1B | 1.57E-31 | | 0.6171042 | | 0.615 | | 0.427 | 4.10E-27 | |
| FKBP4 | 2.23E-41 | | 0.61667506 | | 0.372 | | 0.158 | 5.82E-37 | |
| EEF1B2 | 3.01E-38 | | 0.61288067 | | 0.913 | | 0.867 | 7.88E-34 | |
| VIM | 0.00013005 | | 0.61128746 | | 0.958 | | 0.962 | 1 | |
| RPS19 | 5.22E-60 | | 0.60984644 | | 0.999 | | 0.991 | 1.37E-55 | |
| RPL21 | 4.82E-60 | | 0.60928279 | | 0.989 | | 0.975 | 1.26E-55 | |
| GPBP1 | 6.30E-42 | | 0.60824907 | | 0.782 | | 0.645 | 1.65E-37 | |
| RPL10A | 3.54E-45 | | 0.60351038 | | 0.973 | | 0.956 | 9.26E-41 | |
| HSP90B1 | 2.88E-15 | | 0.60300153 | | 0.642 | | 0.563 | 7.54E-11 | |
| RPL19 | 2.37E-62 | | 0.60222432 | | 0.993 | | 0.991 | 6.19E-58 | |
| UBA52 | 4.08E-57 | | 0.60028524 | | 0.98 | | 0.96 | 1.07E-52 | |
| RPL22L1 | 2.01E-20 | | 0.59938968 | | 0.444 | | 0.3 | 5.26E-16 | |
| H2AC6 | 6.25E-09 | | 0.5984602 | | 0.317 | | 0.23 | 0.00016365 | |
| RPL7A | 1.65E-41 | | 0.59374423 | | 0.987 | | 0.986 | 4.33E-37 | |
| PDE4D | 8.57E-31 | | 0.59280991 | | 0.403 | | 0.221 | 2.24E-26 | |
| RPS14 | 4.99E-60 | | 0.59054749 | | 0.991 | | 0.992 | 1.31E-55 | |
| DNAJB4 | 3.13E-30 | | 0.58883853 | | 0.505 | | 0.315 | 8.20E-26 | |
| TMSB4X | 6.34E-30 | | 0.58843662 | | 0.999 | | 1 | 1.66E-25 | |
| PIM1 | 2.69E-32 | | 0.58285584 | | 0.44 | | 0.253 | 7.04E-28 | |
| RPL11 | 5.64E-57 | | 0.58252452 | | 0.992 | | 0.991 | 1.48E-52 | |
| ZFP36 | 2.66E-13 | | 0.58042817 | | 0.7 | | 0.591 | 6.95E-09 | |
| ATF3 | 1.57E-35 | | 0.57888471 | | 0.345 | | 0.145 | 4.11E-31 | |
| SEC61B | 3.21E-16 | | 0.57636781 | | 0.57 | | 0.459 | 8.41E-12 | |
| PKM | 0.01316385 | | 0.57328522 | | 0.42 | | 0.413 | 1 | |
| SPOCK2 | 1.72E-14 | | 0.57325732 | | 0.571 | | 0.475 | 4.51E-10 | |
| HNRNPA1 | 2.90E-25 | | 0.56986794 | | 0.895 | | 0.823 | 7.58E-21 | |
| DUSP1 | 8.69E-25 | | 0.56926643 | | 0.828 | | 0.655 | 2.27E-20 | |
| CHCHD2 | 5.40E-21 | | 0.56801013 | | 0.784 | | 0.693 | 1.41E-16 | |
| SLC25A5 | 8.04E-33 | | 0.56608072 | | 0.564 | | 0.373 | 2.10E-28 | |
| PPIB | 5.42E-14 | | 0.5639732 | | 0.696 | | 0.619 | 1.42E-09 | |
| LAG3 | 0.00190086 | | 0.56377963 | | 0.383 | | 0.352 | 1 | |
| REL | 3.52E-25 | | 0.55867345 | | 0.552 | | 0.386 | 9.20E-21 | |
| UBB | 3.24E-41 | | 0.55519039 | | 0.944 | | 0.864 | 8.47E-37 | |
| CDKN1A | 5.44E-54 | | 0.54743378 | | 0.395 | | 0.138 | 1.42E-49 | |
| YBX1 | 2.27E-10 | | 0.54576512 | | 0.762 | | 0.709 | 5.93E-06 | |
| NAP1L1 | 1.60E-20 | | 0.54366844 | | 0.745 | | 0.674 | 4.18E-16 | |
| BTG1 | 1.48E-10 | | 0.54216327 | | 0.958 | | 0.972 | 3.88E-06 | |
| KLF6 | 1.02E-13 | | 0.54099698 | | 0.816 | | 0.766 | 2.67E-09 | |
| SELENOK | 4.86E-32 | | 0.54098657 | | 0.61 | | 0.415 | 1.27E-27 | |
| MYL6 | 9.44E-08 | | 0.53954536 | | 0.915 | | 0.905 | 0.00247048 | |
| KLF2 | 5.57E-22 | | 0.53865307 | | 0.467 | | 0.294 | 1.46E-17 | |
| RPS27A | 4.85E-52 | | 0.53757435 | | 0.995 | | 0.994 | 1.27E-47 | |
| MXD1 | 6.38E-43 | | 0.53714581 | | 0.407 | | 0.176 | 1.67E-38 | |
| RPL23A | 9.74E-54 | | 0.53452269 | | 0.994 | | 0.988 | 2.55E-49 | |
| RPS23 | 5.98E-46 | | 0.53422792 | | 0.992 | | 0.984 | 1.56E-41 | |
| SNHG29 | 9.49E-35 | | 0.53234195 | | 0.717 | | 0.537 | 2.48E-30 | |
| TPI1 | 1.57E-09 | | 0.53008791 | | 0.626 | | 0.559 | 4.12E-05 | |
| PTMA | 1.00E-33 | | 0.52753913 | | 0.994 | | 0.991 | 2.62E-29 | |
| SLC2A3 | 2.85E-24 | | 0.52699175 | | 0.677 | | 0.51 | 7.46E-20 | |
| INSIG1 | 2.85E-31 | | 0.52102803 | | 0.251 | | 0.09 | 7.46E-27 | |
| SLC25A6 | 6.34E-29 | | 0.51864577 | | 0.727 | | 0.593 | 1.66E-24 | |
| RPL14 | 5.45E-44 | | 0.51828019 | | 0.991 | | 0.98 | 1.43E-39 | |
| OAZ1 | 3.34E-24 | | 0.51422059 | | 0.858 | | 0.794 | 8.74E-20 | |
| SERPINB9 | 2.28E-24 | | 0.51415771 | | 0.36 | | 0.203 | 5.97E-20 | |
| CLK1 | 5.40E-32 | | 0.51349013 | | 0.724 | | 0.549 | 1.41E-27 | |
| HNRNPC | 4.59E-21 | | 0.50882761 | | 0.77 | | 0.679 | 1.20E-16 | |
| RPS15 | 1.24E-47 | | 0.50823352 | | 0.992 | | 0.996 | 3.25E-43 | |
| BZW1 | 3.85E-20 | | 0.50818085 | | 0.588 | | 0.458 | 1.01E-15 | |
| JUNB | 6.81E-34 | | 0.50620827 | | 0.864 | | 0.66 | 1.78E-29 | |
| IL7R | 5.86E-21 | | 0.50566628 | | 0.717 | | 0.562 | 1.53E-16 | |
| S100A6 | 1.27E-05 | | 0.50521019 | | 0.869 | | 0.877 | 0.33246079 | |
| RPL24 | 1.27E-38 | | 0.50140414 | | 0.981 | | 0.958 | 3.31E-34 | |
| NACA | 6.04E-39 | | 0.50112672 | | 0.977 | | 0.955 | 1.58E-34 | |
| HINT1 | 1.45E-25 | | 0.50001521 | | 0.807 | | 0.708 | 3.78E-21 | |
| TNFRSF18 | 6.57E-15 | | 0.4999714 | | 0.355 | | 0.231 | 1.72E-10 | |
| NR4A2 | 2.29E-21 | | 0.49061863 | | 0.475 | | 0.302 | 5.99E-17 | |
| BTF3 | 1.62E-26 | | 0.48379231 | | 0.89 | | 0.806 | 4.24E-22 | |
| TNFAIP8 | 2.80E-20 | | 0.48332845 | | 0.59 | | 0.446 | 7.33E-16 | |
| TNFSF9 | 4.23E-14 | | 0.48124515 | | 0.269 | | 0.156 | 1.11E-09 | |
| ZFAS1 | 3.64E-29 | | 0.47885187 | | 0.753 | | 0.608 | 9.52E-25 | |
| RPL31 | 2.09E-30 | | 0.4787734 | | 0.952 | | 0.91 | 5.47E-26 | |
| TPM4 | 2.62E-13 | | 0.47738824 | | 0.57 | | 0.467 | 6.86E-09 | |
| ISG20 | 7.04E-19 | | 0.47638116 | | 0.615 | | 0.488 | 1.84E-14 | |
| SEC61G | 9.94E-14 | | 0.47612466 | | 0.48 | | 0.357 | 2.60E-09 | |
| RPL27 | 1.83E-32 | | 0.47610683 | | 0.969 | | 0.927 | 4.80E-28 | |
| PIK3R1 | 6.97E-12 | | 0.47358396 | | 0.609 | | 0.527 | 1.82E-07 | |
| TSPYL2 | 3.73E-16 | | 0.47306284 | | 0.625 | | 0.502 | 9.77E-12 | |
| EFHD2 | 4.30E-18 | | 0.4702871 | | 0.457 | | 0.321 | 1.13E-13 | |
| RPL6 | 3.11E-29 | | 0.46949068 | | 0.988 | | 0.989 | 8.14E-25 | |
| MT-ND2 | 2.02E-09 | | 0.469007 | | 0.941 | | 0.955 | 5.29E-05 | |
| CD55 | 3.88E-37 | | 0.46886036 | | 0.359 | | 0.159 | 1.02E-32 | |
| HERPUD1 | 4.13E-22 | | 0.46828044 | | 0.527 | | 0.373 | 1.08E-17 | |
| GBP5 | 1.63E-12 | | 0.46768289 | | 0.511 | | 0.413 | 4.26E-08 | |
| MT-ATP6 | 3.12E-23 | | 0.46599929 | | 0.995 | | 0.997 | 8.16E-19 | |
| CEBPD | 1.15E-13 | | 0.46599721 | | 0.263 | | 0.157 | 3.01E-09 | |
| UBE2S | 8.42E-25 | | 0.46053547 | | 0.429 | | 0.263 | 2.20E-20 | |
| PRNP | 3.29E-22 | | 0.45885334 | | 0.458 | | 0.3 | 8.60E-18 | |
| LDHB | 2.57E-22 | | 0.45835319 | | 0.647 | | 0.501 | 6.72E-18 | |
| SOCS3 | 1.02E-33 | | 0.45808666 | | 0.396 | | 0.187 | 2.66E-29 | |
| RPLP2 | 2.49E-31 | | 0.45688524 | | 0.994 | | 0.996 | 6.51E-27 | |
| RPL32 | 1.26E-35 | | 0.45397663 | | 0.996 | | 0.997 | 3.29E-31 | |
| BCAS2 | 2.60E-17 | | 0.45240726 | | 0.469 | | 0.334 | 6.79E-13 | |
| EZR | 7.12E-15 | | 0.45230406 | | 0.644 | | 0.554 | 1.86E-10 | |
| NKG7 | 0.00271615 | | 0.45221997 | | 0.685 | | 0.726 | 1 | |
| RPL12 | 5.22E-28 | | 0.4494399 | | 0.992 | | 0.988 | 1.37E-23 | |
| MCL1 | 1.54E-14 | | 0.44591393 | | 0.708 | | 0.643 | 4.04E-10 | |
| NR4A1 | 2.84E-24 | | 0.44565508 | | 0.639 | | 0.442 | 7.42E-20 | |
| TXN | 1.61E-09 | | 0.44401663 | | 0.518 | | 0.433 | 4.22E-05 | |
| MT-ND4 | 0.0020472 | | 0.44315063 | | 0.978 | | 0.991 | 1 | |
| LINC-PINT | 1.10E-16 | | 0.44295028 | | 0.587 | | 0.483 | 2.88E-12 | |
| AHSA1 | 5.39E-30 | | 0.44286476 | | 0.563 | | 0.369 | 1.41E-25 | |
| KDM6B | 4.12E-23 | | 0.44087381 | | 0.328 | | 0.178 | 1.08E-18 | |
| SH2D2A | 2.00E-19 | | 0.44072171 | | 0.347 | | 0.208 | 5.23E-15 | |
| PNRC1 | 9.14E-16 | | 0.43994636 | | 0.844 | | 0.821 | 2.39E-11 | |
| ERN1 | 1.00E-23 | | 0.43855847 | | 0.432 | | 0.255 | 2.63E-19 | |
| TUBB4B | 1.23E-33 | | 0.43837524 | | 0.487 | | 0.275 | 3.22E-29 | |
| ZC3H12A | 6.14E-45 | | 0.43829259 | | 0.326 | | 0.11 | 1.61E-40 | |
| RPS7 | 8.08E-30 | | 0.43819098 | | 0.985 | | 0.986 | 2.11E-25 | |
| RPL36AL | 1.41E-23 | | 0.43570884 | | 0.854 | | 0.747 | 3.69E-19 | |
| H1-2 | 0.51376491 | | 0.43541439 | | 0.374 | | 0.386 | 1 | |
| SLC3A2 | 4.69E-16 | | 0.43435001 | | 0.52 | | 0.398 | 1.23E-11 | |
| ARPC2 | 1.84E-20 | | 0.43329254 | | 0.868 | | 0.801 | 4.83E-16 | |
| STIP1 | 4.45E-26 | | 0.43308134 | | 0.461 | | 0.285 | 1.16E-21 | |
| RAN | 5.01E-11 | | 0.43291773 | | 0.637 | | 0.564 | 1.31E-06 | |
| G3BP2 | 5.35E-15 | | 0.43202138 | | 0.552 | | 0.439 | 1.40E-10 | |
| FAM107B | 3.68E-08 | | 0.4299881 | | 0.648 | | 0.615 | 0.00096326 | |
| ZNF331 | 9.59E-14 | | 0.42896723 | | 0.318 | | 0.201 | 2.51E-09 | |
| PDIA6 | 7.02E-13 | | 0.42870133 | | 0.444 | | 0.336 | 1.84E-08 | |
| SERP1 | 4.17E-23 | | 0.42712066 | | 0.637 | | 0.488 | 1.09E-18 | |
| CD7 | 1.40E-11 | | 0.42672605 | | 0.755 | | 0.662 | 3.66E-07 | |
| COX4I1 | 1.93E-25 | | 0.42555332 | | 0.873 | | 0.785 | 5.06E-21 | |
| RSRP1 | 2.68E-24 | | 0.42440553 | | 0.773 | | 0.692 | 7.02E-20 | |
| TUBB | 4.53E-15 | | 0.42270559 | | 0.398 | | 0.277 | 1.18E-10 | |
| RPS17 | 3.70E-09 | | 0.4225858 | | 0.652 | | 0.598 | 9.69E-05 | |
| CNOT6L | 1.33E-14 | | 0.42239969 | | 0.56 | | 0.449 | 3.48E-10 | |
| HIF1A | 9.06E-07 | | 0.42149988 | | 0.43 | | 0.379 | 0.02369727 | |
| EIF3E | 1.18E-26 | | 0.42144133 | | 0.682 | | 0.51 | 3.08E-22 | |
| CCT3 | 6.52E-25 | | 0.42131178 | | 0.463 | | 0.286 | 1.71E-20 | |
| CHORDC1 | 3.01E-19 | | 0.42046806 | | 0.606 | | 0.456 | 7.87E-15 | |
| RPS25 | 7.86E-34 | | 0.42001485 | | 0.992 | | 0.986 | 2.06E-29 | |
| PRF1 | 0.00090954 | | 0.41993278 | | 0.413 | | 0.39 | 1 | |
| RPL26 | 4.47E-34 | | 0.41948855 | | 0.992 | | 0.991 | 1.17E-29 | |
| PIM3 | 5.04E-23 | | 0.41935754 | | 0.382 | | 0.221 | 1.32E-18 | |
| FXYD5 | 2.06E-25 | | 0.41893251 | | 0.842 | | 0.751 | 5.38E-21 | |
| MRPL18 | 1.17E-21 | | 0.41855428 | | 0.372 | | 0.223 | 3.06E-17 | |
| METRNL | 4.62E-11 | | 0.41795795 | | 0.284 | | 0.19 | 1.21E-06 | |
| EIF3F | 6.35E-28 | | 0.41530257 | | 0.663 | | 0.496 | 1.66E-23 | |
| LDLR | 7.14E-37 | | 0.41421307 | | 0.262 | | 0.083 | 1.87E-32 | |
| BATF | 6.49E-19 | | 0.4139434 | | 0.363 | | 0.221 | 1.70E-14 | |
| PSME2 | 5.04E-11 | | 0.41368013 | | 0.604 | | 0.524 | 1.32E-06 | |
| PGK1 | 6.11E-13 | | 0.41267838 | | 0.568 | | 0.463 | 1.60E-08 | |
| DDX21 | 1.53E-17 | | 0.41038318 | | 0.463 | | 0.325 | 4.00E-13 | |
| UBALD2 | 4.08E-22 | | 0.40911736 | | 0.397 | | 0.244 | 1.07E-17 | |
| TNFSF14 | 4.06E-12 | | 0.40738835 | | 0.413 | | 0.317 | 1.06E-07 | |
| CGAS | 5.80E-20 | | 0.40694185 | | 0.388 | | 0.241 | 1.52E-15 | |
| ATF4 | 4.94E-33 | | 0.40679474 | | 0.431 | | 0.226 | 1.29E-28 | |
| MT-CO3 | 8.46E-11 | | 0.40550709 | | 0.985 | | 0.995 | 2.21E-06 | |
| LITAF | 3.94E-18 | | 0.40251931 | | 0.436 | | 0.3 | 1.03E-13 | |
| NDUFS5 | 7.94E-17 | | 0.40236821 | | 0.655 | | 0.541 | 2.08E-12 | |
| BTG3 | 3.96E-26 | | 0.40228825 | | 0.351 | | 0.182 | 1.04E-21 | |
| MSN | 1.18E-08 | | 0.40148214 | | 0.7 | | 0.656 | 0.0003097 | |
| ANXA1 | 2.20E-14 | | 0.40097654 | | 0.795 | | 0.707 | 5.75E-10 | |
| CALR | 8.84E-07 | | 0.39944779 | | 0.626 | | 0.557 | 0.02312773 | |
| STAT3 | 7.16E-19 | | 0.3982888 | | 0.605 | | 0.482 | 1.87E-14 | |
| CSF1 | 7.75E-06 | | 0.39692283 | | 0.294 | | 0.236 | 0.2027136 | |
| EEF1D | 3.04E-30 | | 0.39628292 | | 0.945 | | 0.89 | 7.96E-26 | |
| EIF5A | 4.96E-05 | | 0.39548952 | | 0.458 | | 0.416 | 1 | |
| NCL | 3.24E-07 | | 0.39535463 | | 0.757 | | 0.729 | 0.00848636 | |
| PTGES3 | 7.70E-11 | | 0.39449058 | | 0.718 | | 0.657 | 2.01E-06 | |
| GNG2 | 1.00E-16 | | 0.39410359 | | 0.478 | | 0.348 | 2.62E-12 | |
| RPS4Y1 | 8.89E-22 | | 0.39363711 | | 0.445 | | 0.265 | 2.33E-17 | |
| CKS2 | 3.25E-24 | | 0.39142441 | | 0.262 | | 0.114 | 8.51E-20 | |
| CD44 | 4.13E-07 | | 0.39122038 | | 0.795 | | 0.783 | 0.0107949 | |
| LMAN1 | 3.65E-15 | | 0.3893268 | | 0.363 | | 0.242 | 9.54E-11 | |
| RGS2 | 4.31E-20 | | 0.38680805 | | 0.757 | | 0.587 | 1.13E-15 | |
| GBP2 | 1.42E-16 | | 0.38645711 | | 0.521 | | 0.392 | 3.71E-12 | |
| MAP3K8 | 1.73E-33 | | 0.38624661 | | 0.27 | | 0.096 | 4.54E-29 | |
| MIAT | 3.92E-16 | | 0.38552211 | | 0.344 | | 0.215 | 1.03E-11 | |
| CHD1 | 7.75E-17 | | 0.38492485 | | 0.452 | | 0.332 | 2.03E-12 | |
| RPS15A | 2.96E-28 | | 0.38435969 | | 0.995 | | 0.996 | 7.75E-24 | |
| CXCR4 | 4.58E-09 | | 0.38379295 | | 0.817 | | 0.786 | 0.00011992 | |
| BCL2A1 | 4.54E-25 | | 0.38298093 | | 0.252 | | 0.105 | 1.19E-20 | |
| GNAS | 2.00E-15 | | 0.3813485 | | 0.784 | | 0.714 | 5.25E-11 | |
| TAF1D | 6.02E-13 | | 0.38134644 | | 0.615 | | 0.52 | 1.57E-08 | |
| PFKFB3 | 8.57E-32 | | 0.38130774 | | 0.275 | | 0.105 | 2.24E-27 | |
| SUB1 | 4.93E-14 | | 0.37842852 | | 0.735 | | 0.643 | 1.29E-09 | |
| SDCBP | 1.10E-23 | | 0.37816141 | | 0.462 | | 0.29 | 2.89E-19 | |
| SNRPB | 1.35E-11 | | 0.37807566 | | 0.584 | | 0.487 | 3.54E-07 | |
| PDIA3 | 4.09E-09 | | 0.37665911 | | 0.62 | | 0.555 | 0.00010712 | |
| MT-CO1 | 0.00945831 | | 0.37599829 | | 0.995 | | 1 | 1 | |
| MOB4 | 4.76E-21 | | 0.37570606 | | 0.345 | | 0.196 | 1.25E-16 | |
| PRRC2C | 3.43E-10 | | 0.37345801 | | 0.801 | | 0.768 | 8.98E-06 | |
| EIF3H | 4.19E-25 | | 0.37218176 | | 0.685 | | 0.527 | 1.10E-20 | |
| CLEC2B | 4.38E-09 | | 0.37142728 | | 0.577 | | 0.507 | 0.00011453 | |
| CDK2AP2 | 5.87E-17 | | 0.36912155 | | 0.414 | | 0.278 | 1.54E-12 | |
| ARPC3 | 8.05E-13 | | 0.36911059 | | 0.794 | | 0.758 | 2.11E-08 | |
| FAM177A1 | 3.47E-26 | | 0.36905091 | | 0.418 | | 0.238 | 9.07E-22 | |
| RPL35 | 1.20E-19 | | 0.36839298 | | 0.981 | | 0.968 | 3.15E-15 | |
| RPL37A | 1.30E-14 | | 0.36826321 | | 0.981 | | 0.978 | 3.39E-10 | |
| SERBP1 | 1.84E-05 | | 0.36705247 | | 0.634 | | 0.591 | 0.48231863 | |
| TNFRSF25 | 2.79E-16 | | 0.36501491 | | 0.343 | | 0.217 | 7.29E-12 | |
| BTG2 | 1.37E-14 | | 0.36498363 | | 0.768 | | 0.646 | 3.57E-10 | |
| PRDX6 | 6.27E-16 | | 0.36497456 | | 0.442 | | 0.313 | 1.64E-11 | |
| MANF | 6.25E-23 | | 0.36426592 | | 0.325 | | 0.172 | 1.64E-18 | |
| SNU13 | 1.08E-17 | | 0.36060346 | | 0.577 | | 0.448 | 2.82E-13 | |
| RHOH | 1.00E-14 | | 0.3605665 | | 0.451 | | 0.33 | 2.62E-10 | |
| ATP5F1B | 2.92E-10 | | 0.35738945 | | 0.479 | | 0.388 | 7.64E-06 | |
| RPL39 | 1.31E-21 | | 0.35729014 | | 0.991 | | 0.99 | 3.42E-17 | |
| TENT5C | 2.73E-19 | | 0.357009 | | 0.384 | | 0.237 | 7.14E-15 | |
| EIF4A2 | 1.07E-20 | | 0.3553725 | | 0.804 | | 0.713 | 2.79E-16 | |
| PSMB4 | 2.47E-17 | | 0.35337679 | | 0.345 | | 0.215 | 6.45E-13 | |
| CCNL1 | 2.54E-14 | | 0.35304756 | | 0.653 | | 0.556 | 6.65E-10 | |
| TAGAP | 5.80E-12 | | 0.35217953 | | 0.456 | | 0.34 | 1.52E-07 | |
| MZT2A | 1.90E-22 | | 0.35193951 | | 0.533 | | 0.363 | 4.96E-18 | |
| SERTAD1 | 1.46E-31 | | 0.3514036 | | 0.354 | | 0.164 | 3.83E-27 | |
| EIF3K | 2.63E-19 | | 0.35017835 | | 0.766 | | 0.654 | 6.87E-15 | |
| FAU | 6.64E-25 | | 0.34998721 | | 0.986 | | 0.986 | 1.74E-20 | |
| PRDX1 | 6.84E-08 | | 0.34984472 | | 0.533 | | 0.467 | 0.00178973 | |
| ANXA2 | 0.0002676 | | 0.34913688 | | 0.516 | | 0.474 | 1 | |
| ISG15 | 3.48E-11 | | 0.34822886 | | 0.534 | | 0.412 | 9.11E-07 | |
| OSTF1 | 3.18E-18 | | 0.3481184 | | 0.498 | | 0.355 | 8.33E-14 | |
| MT-ND4L | 0.06809839 | | 0.34657262 | | 0.533 | | 0.56 | 1 | |
| HNRNPDL | 2.24E-09 | | 0.3457204 | | 0.747 | | 0.693 | 5.86E-05 | |
| PSMB3 | 1.99E-09 | | 0.34506275 | | 0.467 | | 0.382 | 5.21E-05 | |
| H2AZ2 | 1.36E-14 | | 0.34259996 | | 0.586 | | 0.471 | 3.57E-10 | |
| S100A4 | 0.28437516 | | 0.34163173 | | 0.886 | | 0.932 | 1 | |
| COX7B | 2.29E-11 | | 0.34098179 | | 0.547 | | 0.44 | 6.00E-07 | |
| NFKB1 | 1.21E-18 | | 0.34057387 | | 0.331 | | 0.194 | 3.17E-14 | |
| COX5A | 9.81E-13 | | 0.34029624 | | 0.399 | | 0.283 | 2.57E-08 | |
| SF3B5 | 2.81E-12 | | 0.33816561 | | 0.485 | | 0.382 | 7.36E-08 | |
| FOS | 1.63E-09 | | 0.33808805 | | 0.719 | | 0.581 | 4.27E-05 | |
| RHOA | 1.06E-11 | | 0.33782164 | | 0.711 | | 0.627 | 2.76E-07 | |
| H3-3A | 8.83E-14 | | 0.33709575 | | 0.912 | | 0.88 | 2.31E-09 | |
| RBM3 | 9.30E-16 | | 0.33699596 | | 0.651 | | 0.526 | 2.43E-11 | |
| TCP1 | 1.70E-10 | | 0.33599078 | | 0.502 | | 0.406 | 4.46E-06 | |
| SERPINB1 | 8.64E-12 | | 0.3359652 | | 0.349 | | 0.243 | 2.26E-07 | |
| DEDD2 | 3.64E-22 | | 0.33555964 | | 0.505 | | 0.327 | 9.53E-18 | |
| CANX | 8.64E-09 | | 0.33481057 | | 0.461 | | 0.369 | 0.00022598 | |
| FKBP11 | 2.34E-17 | | 0.3347241 | | 0.35 | | 0.216 | 6.11E-13 | |
| RHOG | 4.01E-21 | | 0.33417023 | | 0.427 | | 0.271 | 1.05E-16 | |
| SSR2 | 2.03E-17 | | 0.33347217 | | 0.678 | | 0.551 | 5.32E-13 | |
| XBP1 | 5.79E-23 | | 0.33316235 | | 0.423 | | 0.248 | 1.51E-18 | |
| JAK1 | 2.40E-06 | | 0.33285077 | | 0.72 | | 0.692 | 0.06272139 | |
| CDV3 | 1.73E-08 | | 0.33263642 | | 0.536 | | 0.468 | 0.00045226 | |
| LCP1 | 4.47E-06 | | 0.33182966 | | 0.833 | | 0.829 | 0.11701663 | |
| SNHG8 | 1.27E-18 | | 0.33102168 | | 0.555 | | 0.396 | 3.32E-14 | |
| IRF1 | 2.42E-09 | | 0.33064527 | | 0.708 | | 0.648 | 6.33E-05 | |
| SRSF7 | 1.16E-10 | | 0.33049217 | | 0.833 | | 0.793 | 3.04E-06 | |
| PSMA7 | 1.14E-05 | | 0.32786829 | | 0.683 | | 0.665 | 0.29948038 | |
| TAX1BP1 | 8.09E-11 | | 0.32784878 | | 0.588 | | 0.498 | 2.12E-06 | |
| SOD2 | 8.25E-19 | | 0.32766192 | | 0.391 | | 0.239 | 2.16E-14 | |
| SLC5A3 | 2.01E-11 | | 0.32703169 | | 0.352 | | 0.244 | 5.26E-07 | |
| SEC11C | 3.40E-11 | | 0.32635542 | | 0.347 | | 0.247 | 8.90E-07 | |
| PCBP1 | 6.03E-13 | | 0.32583668 | | 0.579 | | 0.481 | 1.58E-08 | |
| CSRNP1 | 4.05E-29 | | 0.32569915 | | 0.358 | | 0.171 | 1.06E-24 | |
| CCT4 | 2.78E-17 | | 0.32530848 | | 0.54 | | 0.396 | 7.27E-13 | |
| ODC1 | 7.40E-09 | | 0.32489784 | | 0.27 | | 0.187 | 0.00019373 | |
| PPP1R2 | 3.08E-08 | | 0.32412157 | | 0.609 | | 0.537 | 0.00080466 | |
| MAF | 1.28E-05 | | 0.32412157 | | 0.396 | | 0.335 | 0.33470905 | |
| CIB1 | 2.35E-13 | | 0.32195373 | | 0.609 | | 0.515 | 6.14E-09 | |
| CCT2 | 5.31E-16 | | 0.32189461 | | 0.38 | | 0.249 | 1.39E-11 | |
| ADGRE5 | 2.29E-07 | | 0.32135778 | | 0.38 | | 0.31 | 0.00598539 | |
| EML4 | 4.97E-11 | | 0.32073893 | | 0.656 | | 0.592 | 1.30E-06 | |
| SNHG16 | 5.85E-19 | | 0.31870807 | | 0.342 | | 0.199 | 1.53E-14 | |
| HNRNPA2B1 | 7.23E-08 | | 0.31783256 | | 0.927 | | 0.919 | 0.00189128 | |
| SQSTM1 | 1.66E-07 | | 0.3167041 | | 0.484 | | 0.409 | 0.00434685 | |
| MALT1 | 1.01E-14 | | 0.31533193 | | 0.303 | | 0.189 | 2.64E-10 | |
| GADD45A | 3.74E-13 | | 0.31516997 | | 0.292 | | 0.175 | 9.78E-09 | |
| BAZ1A | 2.30E-16 | | 0.315037 | | 0.441 | | 0.31 | 6.02E-12 | |
| CFL1 | 8.60E-05 | | 0.31485544 | | 0.929 | | 0.943 | 1 | |
| CD28 | 8.56E-24 | | 0.31414898 | | 0.285 | | 0.132 | 2.24E-19 | |
| SDF2L1 | 9.27E-12 | | 0.31217536 | | 0.292 | | 0.188 | 2.43E-07 | |
| MT-ND1 | 8.94E-06 | | 0.31203072 | | 0.934 | | 0.952 | 0.23381969 | |
| SMCHD1 | 6.34E-07 | | 0.31154687 | | 0.629 | | 0.575 | 0.01660064 | |
| PFN1 | 0.00099998 | | 0.3107508 | | 0.945 | | 0.957 | 1 | |
| RBM8A | 2.39E-08 | | 0.31027159 | | 0.542 | | 0.479 | 0.00062472 | |
| RPL34 | 2.62E-17 | | 0.31006561 | | 0.995 | | 0.996 | 6.87E-13 | |
| TPM3 | 1.64E-07 | | 0.30978618 | | 0.818 | | 0.757 | 0.00430284 | |
| APRT | 2.94E-09 | | 0.3096945 | | 0.621 | | 0.544 | 7.70E-05 | |
| PFDN5 | 9.70E-20 | | 0.30961706 | | 0.926 | | 0.879 | 2.54E-15 | |
| RPL30 | 1.54E-22 | | 0.30954673 | | 0.994 | | 0.993 | 4.02E-18 | |
| RPS3A | 2.94E-16 | | 0.30950262 | | 0.988 | | 0.983 | 7.68E-12 | |
| CD48 | 4.96E-16 | | 0.30926788 | | 0.829 | | 0.745 | 1.30E-11 | |
| RPL36 | 3.56E-12 | | 0.30704508 | | 0.978 | | 0.97 | 9.32E-08 | |
| DDX3X | 0.00068473 | | 0.30687501 | | 0.677 | | 0.674 | 1 | |
| HSPA4 | 8.25E-15 | | 0.306768 | | 0.314 | | 0.196 | 2.16E-10 | |
| SURF4 | 2.23E-08 | | 0.30527281 | | 0.292 | | 0.214 | 0.00058265 | |
| GPRIN3 | 1.28E-10 | | 0.30526254 | | 0.476 | | 0.378 | 3.34E-06 | |
| ATP5MC2 | 1.07E-12 | | 0.30511923 | | 0.801 | | 0.726 | 2.81E-08 | |
| MYDGF | 4.75E-12 | | 0.30450388 | | 0.276 | | 0.174 | 1.24E-07 | |
| UQCRH | 2.10E-09 | | 0.30363511 | | 0.614 | | 0.527 | 5.51E-05 | |
| RPL22 | 3.98E-14 | | 0.30276457 | | 0.948 | | 0.915 | 1.04E-09 | |
| TKT | 4.64E-13 | | 0.3016938 | | 0.337 | | 0.224 | 1.21E-08 | |
| GOLGB1 | 0.00038156 | | 0.29981046 | | 0.478 | | 0.461 | 1 | |
| GUK1 | 2.07E-12 | | 0.29975201 | | 0.627 | | 0.527 | 5.40E-08 | |
| KMT2E | 0.00170494 | | 0.29841273 | | 0.624 | | 0.608 | 1 | |
| PPP1R15B | 1.22E-16 | | 0.29607775 | | 0.348 | | 0.209 | 3.19E-12 | |
| NFKB2 | 1.79E-16 | | 0.29538322 | | 0.331 | | 0.201 | 4.67E-12 | |
| SSR3 | 6.12E-13 | | 0.29500463 | | 0.4 | | 0.284 | 1.60E-08 | |
| DOK2 | 7.42E-07 | | 0.29431624 | | 0.491 | | 0.426 | 0.01940107 | |
| NDUFA4 | 8.39E-06 | | 0.29315344 | | 0.673 | | 0.632 | 0.21963744 | |
| PRDM1 | 7.95E-06 | | 0.29239787 | | 0.364 | | 0.302 | 0.20788466 | |
| SPTY2D1 | 3.71E-14 | | 0.29218416 | | 0.358 | | 0.234 | 9.70E-10 | |
| RBM39 | 1.21E-08 | | 0.2921406 | | 0.821 | | 0.782 | 0.00031695 | |
| LRRFIP1 | 1.75E-06 | | 0.29175205 | | 0.642 | | 0.607 | 0.04584531 | |
| CALM1 | 7.07E-05 | | 0.29118147 | | 0.939 | | 0.944 | 1 | |
| NOP58 | 8.71E-16 | | 0.29038486 | | 0.44 | | 0.306 | 2.28E-11 | |
| UPP1 | 3.50E-24 | | 0.28960569 | | 0.263 | | 0.113 | 9.17E-20 | |
| PSME1 | 5.44E-10 | | 0.28831739 | | 0.795 | | 0.743 | 1.42E-05 | |
| JPT1 | 4.44E-11 | | 0.2883069 | | 0.415 | | 0.313 | 1.16E-06 | |
| MYL12B | 1.24E-08 | | 0.28809326 | | 0.852 | | 0.843 | 0.00032325 | |
| CYBA | 1.04E-09 | | 0.28632588 | | 0.898 | | 0.889 | 2.71E-05 | |
| YPEL5 | 3.91E-12 | | 0.28496708 | | 0.499 | | 0.39 | 1.02E-07 | |
| GAS5 | 3.26E-13 | | 0.28400218 | | 0.734 | | 0.635 | 8.52E-09 | |
| ANP32B | 1.09E-10 | | 0.28390979 | | 0.625 | | 0.527 | 2.86E-06 | |
| IFNGR1 | 2.08E-14 | | 0.28329555 | | 0.288 | | 0.176 | 5.45E-10 | |
| TAF7 | 5.30E-10 | | 0.2831913 | | 0.652 | | 0.578 | 1.39E-05 | |
| NDUFB8 | 4.90E-08 | | 0.28256908 | | 0.44 | | 0.36 | 0.00128113 | |
| UXT | 4.46E-16 | | 0.28115973 | | 0.575 | | 0.442 | 1.17E-11 | |
| OSTC | 4.00E-11 | | 0.28114289 | | 0.361 | | 0.255 | 1.05E-06 | |
| AC058791.1 | 2.22E-13 | | 0.28093773 | | 0.32 | | 0.203 | 5.80E-09 | |
| PSMA5 | 2.45E-12 | | 0.28086229 | | 0.424 | | 0.309 | 6.41E-08 | |
| RPS24 | 1.06E-12 | | 0.28047237 | | 0.992 | | 0.996 | 2.77E-08 | |
| EDF1 | 1.07E-11 | | 0.28020121 | | 0.725 | | 0.645 | 2.81E-07 | |
| SAP18 | 7.99E-09 | | 0.27991221 | | 0.602 | | 0.525 | 0.00020906 | |
| PRELID1 | 1.72E-11 | | 0.27758926 | | 0.493 | | 0.379 | 4.51E-07 | |
| UBE2D3 | 1.26E-10 | | 0.27529818 | | 0.654 | | 0.569 | 3.29E-06 | |
| FNBP1 | 0.01792393 | | 0.27499935 | | 0.556 | | 0.556 | 1 | |
| RPS21 | 4.71E-08 | | 0.27454511 | | 0.981 | | 0.974 | 0.00123294 | |
| MYBL1 | 1.00E-11 | | 0.2732444 | | 0.271 | | 0.168 | 2.62E-07 | |
| YWHAZ | 1.01E-07 | | 0.2728422 | | 0.807 | | 0.77 | 0.00264631 | |
| PTMS | 0.03127834 | | 0.27274745 | | 0.4 | | 0.377 | 1 | |
| MAP1LC3B | 9.69E-11 | | 0.27221832 | | 0.547 | | 0.448 | 2.54E-06 | |
| NUDC | 2.63E-08 | | 0.27160388 | | 0.414 | | 0.325 | 0.00068919 | |
| CNBP | 1.12E-09 | | 0.27096632 | | 0.65 | | 0.571 | 2.93E-05 | |
| TIMP1 | 1.91E-12 | | 0.27093229 | | 0.309 | | 0.199 | 5.00E-08 | |
| NDUFA12 | 5.48E-13 | | 0.27076245 | | 0.415 | | 0.3 | 1.43E-08 | |
| EIF4A3 | 3.28E-10 | | 0.27063795 | | 0.289 | | 0.195 | 8.58E-06 | |
| SERF2 | 7.70E-06 | | 0.27062592 | | 0.932 | | 0.912 | 0.20147981 | |
| PPP2R5C | 1.63E-12 | | 0.26990988 | | 0.634 | | 0.534 | 4.26E-08 | |
| P4HB | 3.59E-08 | | 0.26936657 | | 0.356 | | 0.272 | 0.0009385 | |
| RPS26 | 0.11595461 | | 0.26766184 | | 0.744 | | 0.735 | 1 | |
| H2AX | 8.06E-16 | | 0.26669329 | | 0.309 | | 0.181 | 2.11E-11 | |
| ACTR3 | 1.55E-05 | | 0.26653408 | | 0.609 | | 0.56 | 0.40561722 | |
| JMJD6 | 8.46E-19 | | 0.26615397 | | 0.289 | | 0.153 | 2.21E-14 | |
| COPA | 6.03E-15 | | 0.26596791 | | 0.374 | | 0.248 | 1.58E-10 | |
| COX6C | 5.27E-06 | | 0.26577758 | | 0.62 | | 0.556 | 0.13792935 | |
| ZC3HAV1 | 1.38E-08 | | 0.26452903 | | 0.456 | | 0.363 | 0.00036167 | |
| SLC25A3 | 2.44E-05 | | 0.2635052 | | 0.5 | | 0.441 | 0.63723442 | |
| CIAO2B | 5.87E-08 | | 0.26309623 | | 0.433 | | 0.352 | 0.00153626 | |
| RPL35A | 3.28E-13 | | 0.2626992 | | 0.986 | | 0.981 | 8.57E-09 | |
| AC016831.4 | 3.30E-19 | | 0.26229425 | | 0.272 | | 0.135 | 8.62E-15 | |
| TANK | 1.16E-12 | | 0.26145229 | | 0.358 | | 0.243 | 3.04E-08 | |
| CCT5 | 2.67E-08 | | 0.26105129 | | 0.326 | | 0.237 | 0.00069781 | |
| TNF | 2.82E-08 | | 0.26063637 | | 0.309 | | 0.216 | 0.00073706 | |
| EIF3I | 7.07E-10 | | 0.26012205 | | 0.387 | | 0.291 | 1.85E-05 | |
| COPE | 9.84E-08 | | 0.25943278 | | 0.496 | | 0.415 | 0.00257324 | |
| TAGLN2 | 3.29E-06 | | 0.25926119 | | 0.682 | | 0.643 | 0.08603594 | |
| NDUFB11 | 5.50E-10 | | 0.25915441 | | 0.53 | | 0.427 | 1.44E-05 | |
| RSRC2 | 3.08E-07 | | 0.25808041 | | 0.577 | | 0.512 | 0.00807029 | |
| PSMB6 | 2.92E-08 | | 0.25794152 | | 0.361 | | 0.279 | 0.0007633 | |
| RNF19A | 5.67E-05 | | 0.25760785 | | 0.56 | | 0.527 | 1 | |
| PLAC8 | 1.87E-17 | | 0.25720443 | | 0.25 | | 0.126 | 4.90E-13 | |
| DYNLL1 | 1.78E-06 | | 0.25713459 | | 0.668 | | 0.615 | 0.0466486 | |
| TIPARP | 4.89E-13 | | 0.25682947 | | 0.319 | | 0.201 | 1.28E-08 | |
| MT-CYB | 0.00620598 | | 0.25624014 | | 0.969 | | 0.995 | 1 | |
| SLC20A1 | 2.22E-09 | | 0.25607189 | | 0.317 | | 0.222 | 5.81E-05 | |
| COX6A1 | 0.00022842 | | 0.25536202 | | 0.642 | | 0.602 | 1 | |
| SNHG7 | 1.46E-15 | | 0.25498758 | | 0.327 | | 0.201 | 3.82E-11 | |
| B2M | 5.57E-10 | | 0.25341722 | | 1 | | 1 | 1.46E-05 | |
| RSL1D1 | 4.38E-12 | | 0.25306541 | | 0.44 | | 0.323 | 1.15E-07 | |
| NABP1 | 3.03E-05 | | 0.25275712 | | 0.444 | | 0.388 | 0.79340292 | |
| EIF5 | 2.86E-12 | | 0.2525091 | | 0.559 | | 0.448 | 7.47E-08 | |
| RAB21 | 8.57E-12 | | 0.25233088 | | 0.37 | | 0.264 | 2.24E-07 | |
| ST13 | 1.96E-09 | | 0.25187218 | | 0.562 | | 0.463 | 5.13E-05 | |
| PRMT1 | 3.30E-06 | | 0.25165658 | | 0.288 | | 0.217 | 0.0864568 | |
| PTPRC | 1.49E-05 | | 0.25050277 | | 0.977 | | 0.981 | 0.39007281 | |
| HLA-DPB1 | 8.05E-16 | | -0.27037599 | | 0.407 | | 0.55 | 2.11E-11 | |
| TRAF3IP3 | 1.58E-18 | | -0.27355199 | | 0.309 | | 0.465 | 4.13E-14 | |
| GZMK | 0.00279907 | | -0.27420241 | | 0.385 | | 0.417 | 1 | |
| HCST | 2.98E-15 | | -0.27522485 | | 0.784 | | 0.87 | 7.80E-11 | |
| CD84 | 4.58E-20 | | -0.27960957 | | 0.175 | | 0.326 | 1.20E-15 | |
| CD8A | 4.08E-16 | | -0.28600916 | | 0.516 | | 0.658 | 1.07E-11 | |
| BTN3A2 | 2.25E-21 | | -0.28865689 | | 0.309 | | 0.48 | 5.89E-17 | |
| GPR174 | 1.90E-24 | | -0.29186081 | | 0.189 | | 0.364 | 4.98E-20 | |
| HLA-DRB1 | 1.66E-10 | | -0.29649581 | | 0.462 | | 0.574 | 4.34E-06 | |
| IKZF3 | 5.35E-18 | | -0.31589369 | | 0.421 | | 0.577 | 1.40E-13 | |
| AHNAK | 1.97E-19 | | -0.31646653 | | 0.626 | | 0.764 | 5.16E-15 | |
| TIGIT | 2.98E-23 | | -0.33111963 | | 0.216 | | 0.398 | 7.79E-19 | |
| RESF1 | 2.12E-25 | | -0.33186689 | | 0.636 | | 0.8 | 5.55E-21 | |
| PTPN7 | 3.38E-16 | | -0.33832789 | | 0.392 | | 0.535 | 8.83E-12 | |
| JAML | 8.77E-12 | | -0.34056033 | | 0.368 | | 0.477 | 2.29E-07 | |
| CD27 | 1.13E-23 | | -0.35525654 | | 0.259 | | 0.437 | 2.97E-19 | |
| CCL5 | 3.51E-16 | | -0.36655267 | | 0.795 | | 0.885 | 9.18E-12 | |
| CD8B | 6.34E-22 | | -0.39743646 | | 0.346 | | 0.52 | 1.66E-17 | |
| TRG-AS1 | 2.66E-23 | | -0.4111781 | | 0.303 | | 0.471 | 6.97E-19 | |
| KRT14 | 1.37E-71 | | -0.44494487 | | 0.014 | | 0.266 | 3.59E-67 | |
| CD3G | 2.36E-27 | | -0.45171129 | | 0.717 | | 0.839 | 6.19E-23 | |
| TTN | 4.77E-36 | | -0.49196464 | | 0.101 | | 0.298 | 1.25E-31 | |
| CRIP1 | 3.22E-42 | | -0.55969273 | | 0.479 | | 0.715 | 8.43E-38 | |
| CD2 | 5.23E-45 | | -0.57049474 | | 0.794 | | 0.904 | 1.37E-40 | |
| CCL4L2 | 3.00E-80 | | -1.03039658 | | 0.116 | | 0.453 | 7.85E-76 | |
|  |  | |  | |  | |  |  | |
| **Supplementary File 1c. Number of Spots after quantification.** | | | | | | | | |  |
| **Samples** | | **Mean_nUMI** | | **Mean_nGene** | | **Total spots** | | |  |
| EOLP | | 18975.35736 | | 3223.513514 | | 666 | | |  |
| NOLP-1 | | 8532.503378 | | 2869.192568 | | 296 | | |  |
| NOLP-2 | | 11239.82887 | | 3119.470238 | | 672 | | |  |
| NOLP-3 | | 16840.56891 | | 3322.493264 | | 965 | | |  |
| Normal-1 | | 14328.00909 | | 3442.531818 | | 440 | | |  |
| Normal-2 | | 15171.08961 | | 3883.52509 | | 558 | | |  |
